# Effects of mesenchymal stromal cell-conditioned media on measures of lung structure and function: a systematic review and meta-analysis of preclinical studies

**DOI:** 10.1186/s13287-020-01900-7

**Published:** 2020-09-15

**Authors:** Alvaro Moreira, Rija Naqvi, Kristen Hall, Chimobi Emukah, John Martinez, Axel Moreira, Evan Dittmar, Sarah Zoretic, Mary Evans, Delanie Moses, Shamimunisa Mustafa

**Affiliations:** 1 Department of Pediatrics, Division of Neonatology, University of Texas Health Science-San Antonio, San Antonio, TX 78229-3900 USA; 2 Department of Pediatrics, Division of Critical Care, Baylor College of Medicine, Houston, TX USA

**Keywords:** Conditioned media, Mesenchymal stem cell, Lung disease, Animal, Review

## Abstract

**Background:**

Lung disease is a leading cause of morbidity and mortality. A breach in the lung alveolar-epithelial barrier and impairment in lung function are hallmarks of acute and chronic pulmonary illness. This review is part two of our previous work. In part 1, we demonstrated that CdM is as effective as MSCs in modulating inflammation. Herein, we investigated the effects of mesenchymal stromal cell (MSC)-conditioned media (CdM) on (i) lung architecture/function in animal models mimicking human lung disease, and (ii) performed a head-to-head comparison of CdM to MSCs.

**Methods:**

Adhering to the animal Systematic Review Centre for Laboratory animal Experimentation protocol, we conducted a search of English articles in five medical databases. Two independent investigators collected information regarding lung: alveolarization, vasculogenesis, permeability, histologic injury, compliance, and measures of right ventricular hypertrophy and right pulmonary pressure. Meta-analysis was performed to generate random effect size using standardized mean difference with 95% confidence interval.

**Results:**

A total of 29 studies met inclusion. Lung diseases included bronchopulmonary dysplasia, asthma, pulmonary hypertension, acute respiratory distress syndrome, chronic obstructive pulmonary disease, and pulmonary fibrosis. CdM improved all measures of lung structure and function. Moreover, no statistical difference was observed in any of the lung measures between MSCs and CdM.

**Conclusions:**

In this meta-analysis of animal models recapitulating human lung disease, CdM improved lung structure and function and had an effect size comparable to MSCs.

## Background

Pulmonary illness is a leading cause of morbidity and mortality [1]. In children, acute respiratory exacerbations are a common reason for primary care visits and are often implicated in hospitalizations [2, 3]. Many of these pulmonary conditions result in impairments in lung function that may last into adulthood [4, 5]. Consequently, identifying novel therapies for lung disease is highly warranted.

A unifying theme in many lung diseases includes inflammation [6–8]. While some inflammation is necessary to combat new disease and for proper wound healing, chronic inflammation may result in altered lung structure and function. During an acute illness, current therapies focus on restoring lung function by abating inflammation [9–11]. For instance, glucocorticoids are the mainstay therapy for reducing inflammation during acute exacerbations of asthma [12]. More recently, mesenchymal stromal/stem cells (MSCs) have shown encouraging outcomes in animal models of lung inflammation [13–15].

MSCs are promising agents as they are easily harvested, can be rapidly expanded, and can secrete factors (exosomes, microvesicles, microRNA) known to reduce inflammation [16–18]. The “secretome” or “conditioned media” of MSCs is considered biologically active and can be easily collected from the surrounding fluid of propagating cells [19–21]. Remarkably, preclinical studies suggest MSC conditioned media (CdM) may be as restorative as the MSCs themselves [22, 23]. We supported this observation in a previous systematic review and meta-analysis demonstrating that CdM is as effective as MSCs in modulating inflammation [24].

This review is an extension of our previous work. In this review, we examined the effects of CdM on (i) lung architecture/function in animal models recapitulating lung disease and (ii) compare these findings to MSCs. Given that the therapeutic benefit of MSCs is attributed to a paracrine fashion, we believed CdM would have comparable effects to MSCs.

## Methods

### Overview and literature search

The methods in our review abide to those outlined by the Systematic Review Centre for Laboratory Animal Experimentation (SYRCLE) [25]. Our protocol was registered through the Collaborative Approach to Meta-Analysis and Review of Data from Experimental Studies (CAMARADES) [26]. Details are described in our previous publication.

We conducted a literature search in five databases using the following terms: mesenchymal stem cell-conditioned media, lung disease, and animal. The last search was performed on March 17th, 2020. Three independent investigators evaluated titles and abstracts, followed by full-text review.

### Inclusion criteria and outcomes of interest

We included studies administering MSC-CdM to animal models of acute lung injury or acute respiratory distress syndrome (ALI/ARDS), asthma, bronchopulmonary dysplasia (BPD), chronic obstructive pulmonary disease (COPD), cystic fibrosis (CF), pneumonia, pulmonary fibrosis (PF), and pulmonary hypertension (PH). Refer to Supplementary File 1 for the list of included studies.

### Outcomes of interest

Measures of lung structure and/or function were our primary endpoint. Lung architecture and function were assessed under the following categories: alveolarization, vasculogenesis, right ventricular hypertrophy, fibrosis, permeability, pulmonary pressures, compliance, and lung injury. Although the pathogenesis of the included lung diseases are heterogeneous, we combined all processes irrespective of disease. This was conducted to obtain a scoping overview of the impact of CdM on biologic processes implicated in lung disease. Subsequently, we assessed lung structure/function by disease in our subgroup analysis. Excluded studies were those which did not provide data concerning our primary outcome of inflammation.

### Data extraction

Three groups of investigators were used (ED and CE; RN and JM; ME, DM, and SM) to collect data. Uniformity of data was assessed by the primary author. This data included general study design, animal model characteristics, conditioned media characteristics, and outcomes of interest.

### Data analysis

A random effects model was used to generate forest plots. A minimum of three studies were required for each outcome to proceed with a meta-analysis. The estimated effect size of CdM or MSC on lung architecture/function was determined using standardized mean difference (SMD) with a 95% confidence interval (CI). Statistical heterogeneity between studies was calculated using the *I*^2^ metric, and funnel plots were used to examine publication bias. If more than six articles were included per outcome, we conducted a subgroup analysis for disease, animal species, and route and dose of CdM administration. All statistical analyses were performed in R version 3.6.2; packages used included *dmetar, metafor*, and *meta*.

## Results

### Study selection

Our literature search resulted in 245 articles. After removing duplicates and viewing the titles and abstracts, 55 articles underwent full-text review. Twenty-nine articles met inclusion (refer to Supplementary Figure 1).

### Study details

Table 1 summarizes the relevant study characteristics. Articles included in the review were published between the years 2009 to 2020. BPD was the most common animal model (*n* = 8), followed by ALI/ARDS (*n* = 5) and asthma (*n* = 5). All of the studies used rodents to induce their lung model.

**Table 1:** Detailed summary of information extracted from included studies

No.	Author (year)	Study design		Animal characteristics		Intervention characteristics		Outcomes
		Disease model	Disease induction	Animal model Gender	Age	Source; (Origin)	Dose; delivery; timing; frequency	Lung architecture/function
1	Ahmadi (2016)	Asthma	Ovalbumin	Wistar rats Male	Adult	Bone marrow	50 μl; IV; 1-day post sensitization; × 1	Tracheal reactivity
2	Ahmadi (2017)	Asthma	Ovalbumin	Wistar rats Male	Adult	Bone marrow	50 μl; IT; 1-day post sensitization; × 1	Histologic lung injury
3	Aslam (2009)	BPD	Hyperoxia	FVB mice Mixed	Neonate	Bone marrow	50 μl; IV; postnatal day 4; × 1	Alveolarization RVH Vasculogenesis
4	Chailakhyan (2014)	ALI	LPS from *E. coli*	Wistar rats Male	NR	Bone marrow	1000 μl; IV; 1 h after LPS injection; × 1	Histologic lung injury
5	Chaubey (2018)	BPD	Hyperoxia	C57BL/6 mice NR	Neonate	Human umbilical cord tissue	100 μl; IP; PN2 and PN4; × 1	Alveolarization RVH Pulmonary artery pressure
6	Cruz (2015)	Asthma	*Aspergillus fumigatus* sensitization	C57/BL6 mice Male	Adult	Bone marrow	200 μl; IV; 14 days after *Aspergillus* challenge; × 1	Histologic lung injury
7	Curley (2013)	ALI/ ARDS	High stretch mechanical ventilation	Sprague–Dawley rats (pathogen-free) Male	Adult	Bone marrow	300 μl; IT; 2.5–3 h post injury initiation; × 1	Alveolarization Histologic lung injury Compliance Wet, dry lung weight ratios Blood gas
8	Felix (2020)	PF	Bleomycin	Wistar rats NR	Adult	Adipose tissue	200 μl; IV; 10 days after induction; × 1	Histologic lung injury Fibrosis
9	Gülaşı (2015)	BPD	Hyperoxia	Wistar rats Mixed	Neonate	Bone marrow	25 μl; IT; on the 11th day; at every inspiration; × 1	Alveolarization
10	Hansmann (2012)	BPD	Hyperoxia	FVB mice Mixed	Adult	Bone marrow	50 μl; IV postnatal day 14; × 1	Alveolarization Fibrosis Compliance/Resistance
11	Hayes (2015)	VILI/ALI	Ventilator-induced	Sprague–Dawley rats Male	Adult	Bone marrow	500 μl; 1.5 h after injury; × 1	Alveolarization Permeability Compliance
12	Huh (2011)	Emphysema (COPD)	Cigarette smoke-induced	Lewis rats Female	Adult	Bone marrow	300 μl; IV; 6 months of age; × 10	Alveolarization Vascularization Pulmonary artery pressure
13	Hwang (2016)	LIRI	Left lung was clamped, re-ventilated, and perfused	Sprague–Dawley rats Male	Adult	Bone marrow	200 μl, IT, 30 min prior to disease induction; × 1	Permeability
14	Ionescu (2012)	ARDS	LPS from *E. coli*	C57/BL6 mice Male	Adult	Bone marrow	30 μl; IT; 4 h post-LPS exposure; × 1	Permeability Histologic lung injury
15	Kennelly (2016)	COPD	Receptor knockout	NOD-SCID IL-2rg^null^ Mice NR	NR	Human bone marrow	IN, day 0 + 6 h; × 2	Alveolarization
16	Keyhanmanesh (2018)	Asthma	Ovalbumin	Wistar rats Male	Adult	Bone marrow	50 μl; IV, single dose, day 33; repeated dose days 33–35	Histologic lung injury
17	Li (2018)	PF	Silica	Wistar rats Female	Adult	Bone marrow	1 mL, IT, days 1 and 4 post-silica; × 2	Fibrosis Histologic lung injury
18	Lu (2015)	ARDS	LPS from *E. coli*	C57/BL6 mice Male	NR	Adipose tissue	200 μl; IV; 4 h post-LPS exposure; × 1	Permeability
19	Pierro (2012)	BPD	Hyperoxia	Newborn rats Mixed	Neonate	Human umbilical cord blood	7 μl/g; IP; postnatal day 4–21 (prevention studies) or from postnatal day 14–28 (regeneration studies); × 18 vs. × 15	Alveolarization Vascularization RVH Compliance Exercise capacity
20	Rahbarghazi (2019)	Asthma	Ovalbumin	Wistar rats Male	Adult	Bone marrow	50 μl; IT; day 33; × 1	Histologic lung injury
21	Rathinasabapathy (2016)	PH	Monocrotaline	Sprague–Dawley rats Male	Adult	Adipose tissue	100 μl; IV; 14 days post-MCT exposure; × 1	Vasculogenesis RVH Fibrosis
22	Sadeghi (2019)	SM	CEES	C57/BL6 mice Male	6–8 weeks	Adipose tissue	500 μl; IP; start week 28; × 8	Fibrosis
23	Shen (2014)	PF	Bleomycin	Wistar rats Female	NR	Bone marrow	200 μl; IT; at 6 h and on day 3 following disease induction; × 2	Fibrosis
24	Su (2019)	ALI	LPS from *E. coli*	C57BL/6 mice Male	8–12 weeks old	NR	200 μl; IV; 4 h after disease induction; × 1	Lung injury
25	Sutsko (2012)	BPD	Hyperoxia	Sprague–Dawley rats Mixed	Neonate	Bone marrow	50 μl; IT; postnatal day 9; × 1	Alveolarization Vascularization RVH
26	Tropea (2012)	BPD	Hyperoxia	FVB mice NR	Neonate	Bone marrow	50 μl; IV; postnatal day 4; × 1	Alveolarization
27	Wakayama (2015)	ARDS	Bleomycin	C57/BL6J mice Female	Adult	Human exfoliated deciduous teeth	500 μl; IV; 24 h post-bleomycin exposure; × 1	Fibrosis
28	Waszak (2012)	BPD	Hyperoxia	Sprague–Dawley rats Mixed	Neonate	Bone marrow	1 μl/g; IP; postnatal day 0 to postnatal day 21; × 22	Alveolarization Vasculogenesis RVH Pulmonary artery pressure
29	Zhao (2014)	Bronchiolitis obliterans	Transplanted donor trachea	C57BL/6 mice Male	Adult	Placenta derived	Volume NR; IT; 3rd day after transplantation; × 1	Tracheal luminal obliteration

*ALI* acute lung injury, *ARDS* acute respiratory distress syndrome, *BPD* bronchopulmonary dysplasia, *CEES-2* chloroehtyl ethyl sulfide, *COPD* chronic obstructive pulmonary disease, *IP* intraperitoneal, *IT* intratracheal, *IV* intravenous, *LIRI* lung ischemia reperfusion injury, *LPS* lipopolysaccharide, *MCT* monocrotaline, *NR* not reported, *PF* pulmonary fibrosis, *RVH* right ventricular hypertrophy, *SM* sulfur mustard chemical lung injury, *VILI* ventilator-induced lung injury

### CdM characteristics

Conditioned media properties are summarized in Supplementary File 3. Stem cells were most isolated from bone marrows and cultured in Dulbecco’s modified Eagle’s medium. Incubation time of the CdM ranged from 24 to 72 h. The volume of CdM administered ranged from 25 μl to 1 ml.

### Alveolarization

**Fig. 1:**
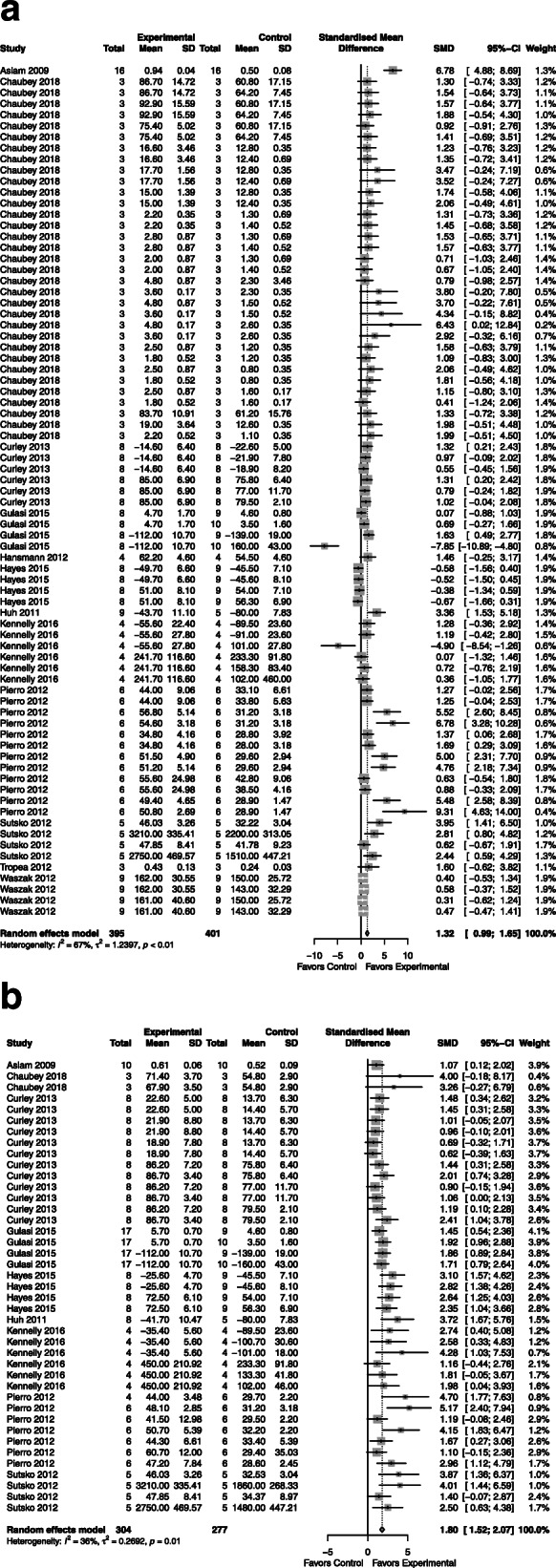
Effect size of CdM (**a**) and MSC (**b**) on lung alveolarization. Forest plots demonstrate SMD with 95% confidence interval

- *CdM*: improved alveolarization with an SMD of 1.32 (95% CI 0.99, 1.65; 12 studies; Fig. 1a) with moderate heterogeneity (*I*^2^ = 67%; *p* < 0.01).
- *MSC*: improved alveolarization with an SMD of 1.80 (95% CI 1.52, 2.07; 9 studies; Fig. 1b) with mild heterogeneity between groups (*I*^2^ = 36%; *p* = 0.01).
- *CdM* vs. *MSC*: no significant difference (Supplementary Figure 2).

### Right ventricular hypertrophy

**Fig. 2:**
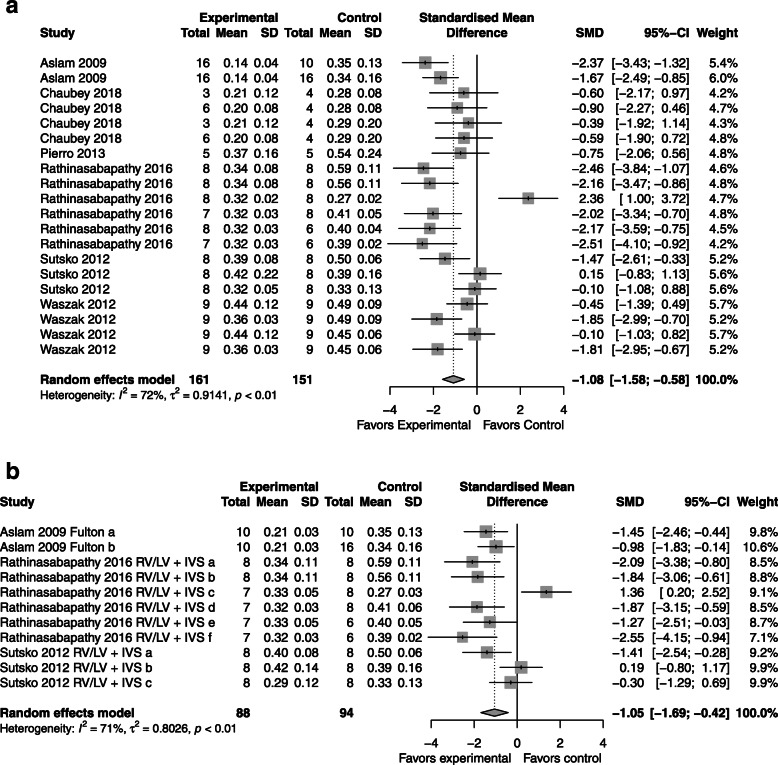
Effect size of CdM (**a**) and MSC (**b**) on right ventricular hypertrophy. Forest plots demonstrate SMD with 95% confidence interval

- *CdM*: favored CdM over control with an SMD of − 1.08 (95% CI − 1.56, − 0.61); 6 studies; Fig. 2a) with significant heterogeneity (*I*^2^ = 70%; *p* < 0.01).
- *MSC*: favored over the control with an SMD of − 1.05 (95% CI − 1.69, − 0.42; 3 studies, Fig. 2b) with significant heterogeneity between groups (*I*^2^ = 71%; *p* < 0.01).
- *CdM* vs. *MSC*: no significant difference (SMD − 0.22, 95% CI − 0.36, 0.16; Supplementary Figure 3).

### Lung fibrosis

**Fig. 3:**
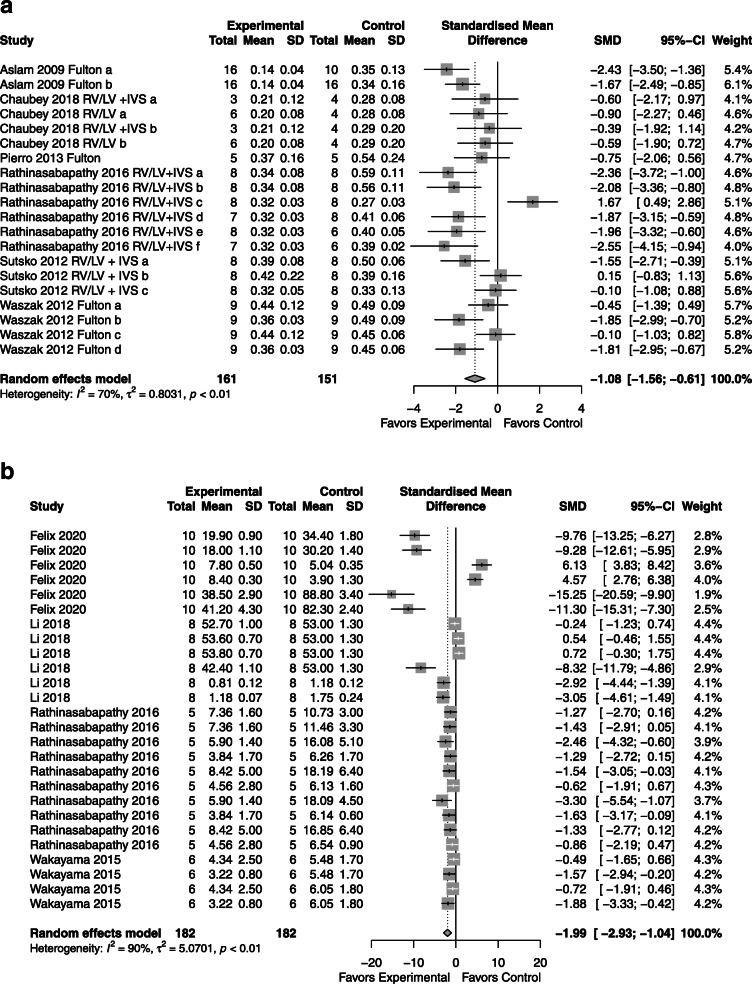
Effect size of CdM (**a**) and MSC (**b**) on lung fibrosis. Forest plots demonstrate SMD with 95% confidence interval

- *CdM*: favored CdM over control with an SMD of − 1.08 (95% CI − 1.56, − 0.61; 6 studies; Fig. 3a) with significant heterogeneity (*I*^2^ = 70%; *p* < 0.01).
- *MSC*: favored MSC over the control with an SMD of − 1.99 (95% CI − 2.93, − 1.04; 4 studies; Fig. 3b) with significant heterogeneity between groups (*I*^2^ = 90%; *p* < 0.01).
- *CdM* vs. *MSC*: the comparison between CdM and MSCs was similar (refer to Supplementary Figure 4).

### Vasculogenesis

**Fig. 4:**
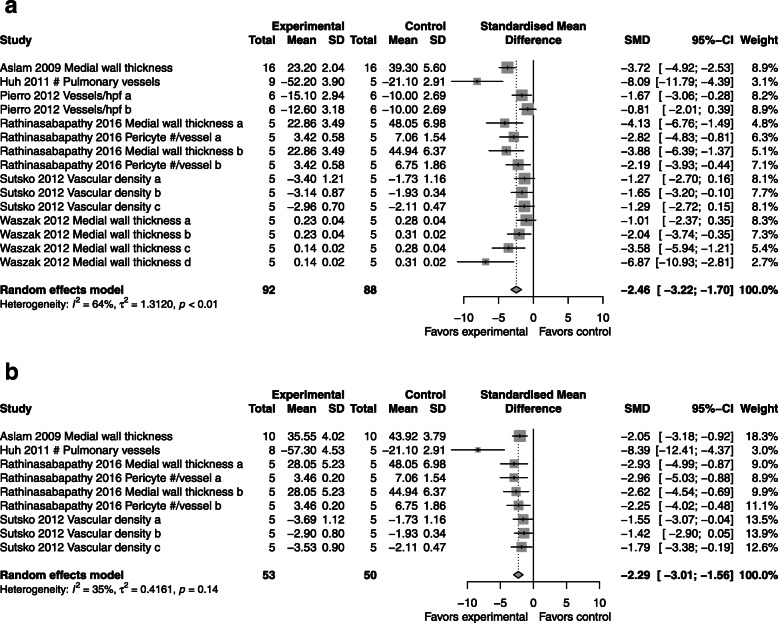
Effect size of CdM (**a**) and MSC (**b**) on lung vascularization. Forest plots demonstrate SMD with 95% confidence interval

- *CdM*: superior to control with an SMD of − 2.46 (95% CI − 3.22, − 1.70; 6 studies; Fig. 4a) with moderate heterogeneity (*I*^2^ = 76%; *p* < 0.01).
- *MSC*: superior to control with an SMD of − 2.29 (95% CI -3.01, − 1.56; 4 studies; Fig. 4b) with mild heterogeneity between groups (*I*^2^ = 35%; *p* = 0.14).
- *CdM* vs. *MSC*: overall effectiveness between CdM and MSCs again showed no significant difference (Supplementary Figure 5).

### Permeability

**Fig. 5:**
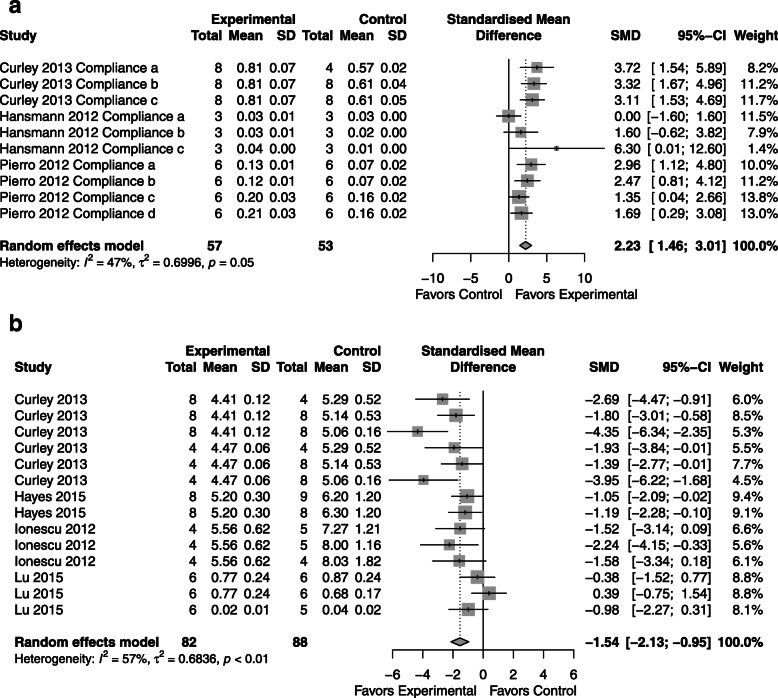
Effect size of CdM (**a**) and MSC (**b**) on lung permeability. Forest plots demonstrate SMD with 95% confidence interval

- *CdM*: permeability assessment favored CdM over control with an SMD of − 0.99 (95% CI − 1.32, − 0.66; 5 studies; Fig. 5a) homogeneity that is non-significant (*I*^2^ = 11.0%; *p* = 0.33).
- *MSC*: in the evaluation of permeability, the MSC was favored over the control with an effect size of − 1.54 (95% CI -2.13, − 0.95; 4 studies; Fig. 5b) with heterogeneity between groups (*I*^2^ = 57.0%; *p* < 0.01).
- *CdM* vs. *MSC*: equal effectiveness (Supplementary Figure 6).

### Pulmonary pressures

**Fig. 6:**
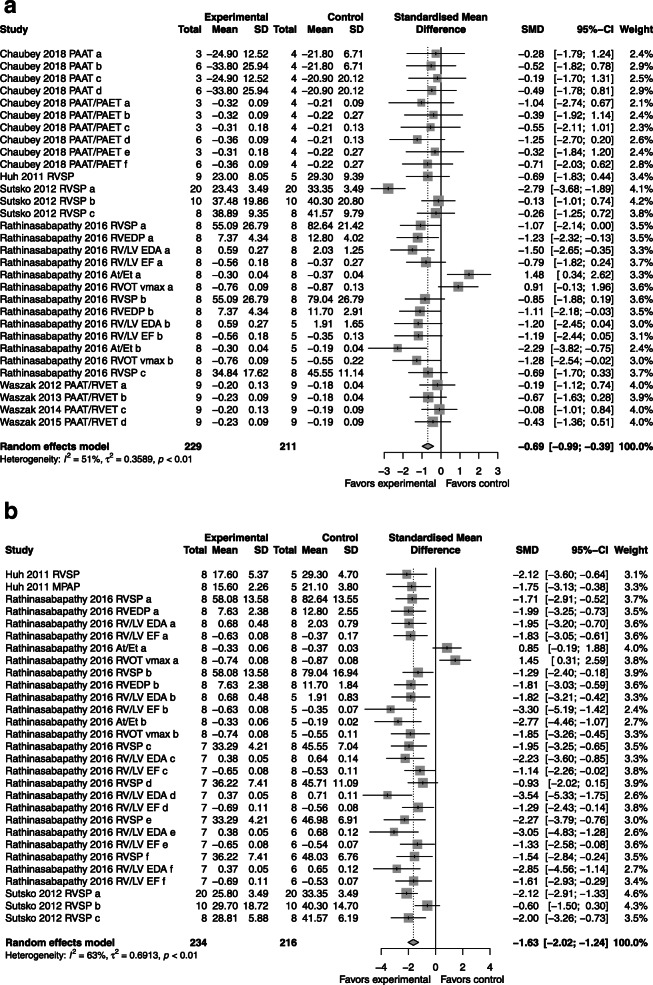
Effect size of CdM (**a**) and MSC (**b**) on pulmonary pressures. Forest plots demonstrate SMD with 95% confidence interval

- *CdM*: improvement in right ventricular pressures compared to control with an SMD of − 0.69 (95% CI − 0.99, − 0.39; 5 studies; Fig. 6a) with moderate heterogeneity (*I*^2^ = 51%; *p* < 0.01).
- *MSC*: superior to control with an SMD of − 1.63 (95% CI − 2.02, − 1.24; 3 studies; Fig. 6b) with moderate heterogeneity (*I*^2^ = 63%; *p* < 0.01).
- *CdM* vs. *MSC*: comparable (please refer to Supplementary Figure 7).

### Histologic lung injury

**Fig. 7:**
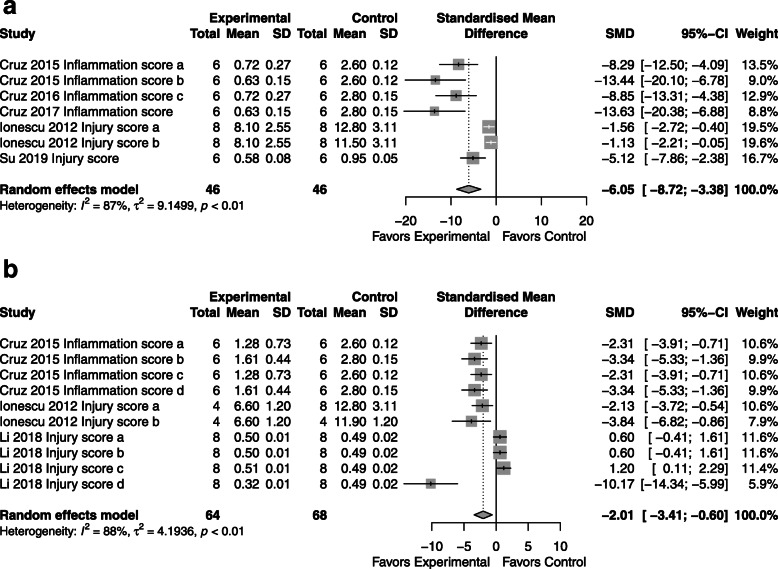
Effect size of CdM (**a**) and MSC (**b**) on histologic lung injury. Forest plots demonstrate SMD with 95% confidence interval

- *CdM*: improvement in histologic lung injury compared to control with an SMD of − 6.05 (95% CI − 8.72, − 3.38; 3 studies; Fig. 7a) with significant heterogeneity (*I*^2^ = 87%; *p* < 0.01).
- *MSC*: superior to control with an SMD of − 2.01 (95% CI -3.41, − 0.60; 3 studies; Fig. 7b) with significant heterogeneity (*I*^2^ = 88%; *p* < 0.01).
- *CdM* vs. *MSC*: less than 3 studies; comparison not performed.

### Compliance

**Fig. 8:**
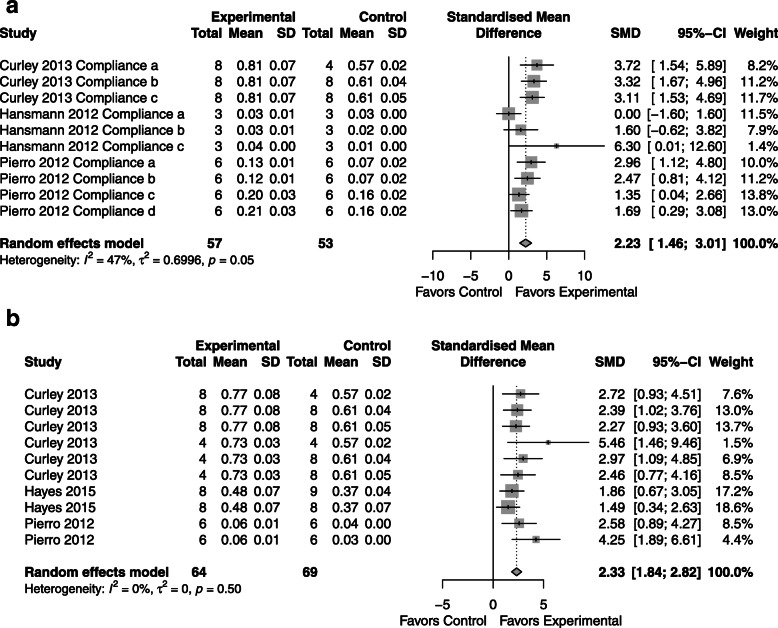
Effect size of CdM (**a**) and MSC (**b**) on pulmonary compliance. Forest plots demonstrate SMD with 95% confidence interval

- *CdM*: improvement in lung compliance compared to control with an SMD of 1.75 (95% CI 0.81, 2.69; 4 studies; Fig. 8a) with significant heterogeneity (*I*^2^ = 76%; p < 0.01).
- *MSC*: improvement in lung compliance compared to control with an SMD of 2.33 (95% CI 1.84, 2.82; 3 studies; Fig. 8b) with no heterogeneity (*I*^2^ = 0%; *p* = 0.5).
- *CdM* vs. *MSC*: not applicable as less than three studies performed a head-to-head comparison.

### All outcomes for lung structure and function combined

- *CdM*: Supplementary Figure 8A shows the SMD of − 1.38 (with 95% CI of − 1.57, − 1.19) favoring CdM over control.
- *MSC*: Supplementary Figure 8B shows the SMD of − 1.66 (with 95% CI of − 1.91, − 1.41) favoring MSC over control.
- *CdM* vs. *MSC*: no difference was appreciated between CdM and MSC when all outcomes were combined (Supplementary Figure 8C).

### Subgroup analysis

Stratification of data was performed by lung disease, tissue source, dose, and route of delivery of CdM. Evaluation was performed if more than 6 studies had data.

#### Alveolarization

Supplementary Figure 9A–D demonstrates that CdM had the greatest impact on alveolarization in BPD animal models (SMD 1.67) and when the media was derived from cord blood (SMD 2.89), given at a dose of 7 μl/g (SMD 2.89), and delivered via the intraperitoneal route (SMD 1.56).

#### RVH

Supplementary Figure 10A–D depicts that CdM significantly improved RVH in BPD animal models (SMD − 0.93) and only when the media was derived from adipose tissue (SMD − 1.05), given at a dose of 100 μl (SMD − 1.14) and delivered intravenously (SMD − 0.86).

#### Fibrosis

Supplementary Figure 11A–D illustrates that CdM had the greatest impact in animal models of BPD and PH (SMD − 4.1, − 3.4, respectively) and when the media was derived from adipose tissue (SMD − 2.61), given at a dose of 50 μl (SMD − 4.10) and delivered intravenously (SMD − 1.95).

#### Vascularization

Supplementary Figure 12A–D shows that CdM had the greatest impact in animal models of COPD (SMD − 8.09), when the media was derived from adipose tissue (SMD − 2.61), given at a dose of 300 μl (SMD − 8.09) and delivered intravenously (SMD − 3.65).

### Risk of bias

No study was judged as low risk across all ten domains. Eight studies stated that the allocation selection was random. Most studies (*n* = 25) had similar groups at baseline. Risk of bias was large regarding allocation concealment, whether authors mention random housing of animals, and blinding of caregivers or random selection of outcome. All studies were found to sufficiently report complete data and being free from other bias. Refer to Supplementary File 2 [27].

### Publication bias

Supplementary Figures 13, 14, 15, 16, 17, 18, 19, and 20 illustrate publication bias through funnel plots. Overall, publication bias was low in all the outcomes except for lung permeability.

## Discussion

Preclinical studies reiterate the ability MSCs have on dampening lung inflammation. This capacity is largely due to the paracrine secretion of MSC factors (microvesicles, exosomes) that provide a basis for future cell-free therapies for human disease [28–31]. This is the first review to directly compare the effects of CdM vs MSCs on lung structure and function in animal models of diverse lung disease. Overall, we found that CdM improved measures of alveolarization, right ventricular hypertrophy, lung fibrosis, vasculogenesis and permeability. Furthermore, CdM reduced pulmonary pressures, ameliorated histologic lung injury, and increased lung compliance. We found that CdM was comparable to MSCs in all lung measures evaluated individually and when combined.

The bioactive factors contained in the CdM of MSCs have been the focus of multiple studies and review articles [32–34]. Congruent with the findings found in this review, Hansmann et al. show that MSC-CdM, compared to CdM from lung fibroblasts, reversed alveolar injury, normalized lung function (airway resistance), and reversed RVH [35]. Additionally, the same group recently demonstrated that MSC exosomes (molecular cargo found within CdM) restored lung architecture, stimulated pulmonary blood vessel formation, and modulated lung inflammation [22]. In an *E. coli* pneumonia-induced ALI mouse model, MSC microvesicles (also found in MSC-CdM) reduced lung permeability and histologic injury score and were equivalent to MSCs [36]. Together, these findings, and those in recent reviews, substantiate the results found in this review [37, 38].

This year, Augustine et al. published a network meta-analysis comparing stem cell and cell-free therapies in preclinical measures of BPD. MSC-CdM had a similar effect size to MSCs regarding alveolarization (MSC SMD 1.71 vs. CdM SMD1.68), angiogenesis (SMD 2.24 vs. 1.79), and pulmonary remodeling (1.29 vs. 1.22) [39]. Similar to their results, this review showed that CdM had among the largest impact on measures of alveolarization and vasculogenesis, processes critical for appropriate lung healing, development, and function [40]. Although vasculogenesis/angiogenesis is an important process to restore lung function/structure, it can also enhance remodeling and thus worsen outcomes in other lung diseases such as asthma or pulmonary fibrosis [41]. In Supplementary Figure 12A, we demonstrate that this process improved in BPD, pulmonary hypertension, and COPD but was not assessed in asthma/pulmonary fibrosis.

In the study by Hayes et al., they found that MSCs were superior to CdM in a rodent model of ventilator-induced lung injury. However, our review suggests that when you compile the literature, there were no significant benefits of using cells over CdM. We cannot explain why CdM was not comparable in this study; however, an important challenge that remains in the field includes the rigorous testing of key variables (tissue source, dose, route, disease, etc.) that may impact the quality of CdM [42–44]. For instance, we found that the intravenous route provided optimal results. Moreover, multiple administrations of CdM may augment vascular development, as seen in the study by Huh et al (*n* = 10 intravenous injections). Conversely, the optimal source and dose of CdM is dependent on the variable or the lung disease. This brings to light that it will be incredibly challenging to find a single CdM product that is ideal for all lung diseases. Thus, the idea of “one-size-fits-all” does not hold true for regenerative cells or products. Illustrating this concept, Rathinasabapathy et al. showed greater improvement in measures of RVH compared to other studies measuring right ventricular size. Important differences seen in the study by Rathinasabapathy and colleagues was that they used a different animal model (PH vs. BPD) and age of rodents (adult vs. neonatal) [45].

As investigators, we should attempt to tease out these characteristics in order to have the ideal product(s) for our lung disease of interest. In this way, we may have translational success in future clinical studies. Refining these features will take time but will play a vital role in efficacy. Moreover, pinpointing small and large animal models of lung disease that will recapitulate what occurs at the patient bedside is essential if we want to move the needle in the field [46].

The plausibility of using a cell-free product as a therapeutic agent for lung disease is substantiated by newly registered human clinical trials. For instance, NCT04235296 and NCT04234750 are evaluating safety of MSC-CdM in regulating wound inflammation and promoting wound healing in burn injury. Another Phase I trial (NCT04134676) plans to study the therapeutic potential of umbilical cord tissue-derived stem cell CdM on chronic skin ulcers. Trials valuing the safety of stem cell CdM constituents (exosomes) are also underway for ischemic stroke (NCT3384433) and ocular conditions (NCT04213248, NCT03437759).

There are several limitations to our systematic review and meta-analysis, many of which mirror those published in our previous report. We incorporated multiple animal models of lung disease that have diverse pathologic processes resulting in their etiology. Also, most of the studies lacked methodologic details rendering them with an unclear risk of bias. Moreover, although preclinical models of lung disease have been helpful in identifying targetable mechanisms/processes, they oftentimes lack the intricacies of human disease. Thus, meticulous efficacy studies in large animals may be one approach to mitigate translational failure in human trials.

## Conclusion

This review demonstrates that the administration of CdM in animal models of lung disease improves lung architecture and function. When compared to MSCs, CdM is as efficacious and provides a basis that cell-free products are a viable option for future studies. However, mores studies are needed to identify how specific variables (tissue source, route of delivery, concentration, etc.) may impact/strengthen their therapeutic potential.

## Supplementary information

**Additional file 1: Figure S1.** Flow diagram demonstrating study selection process.

**Additional file 2: Figure S2.** Effect size of CdM vs. MSC on lung alveolarization. . Forest plots demonstrate SMD with 95% confidence interval.

**Additional file 3: Figure S3.** Effect size of CdM on right ventricular hypertrophy. Forest plots demonstrate SMD with 95% confidence interval.

**Additional file 4: Figure S4.** Effect size of MSC on lung fibrosis. Forest plots demonstrate SMD with 95% confidence interval.

**Additional file 5: Figure S5.** Effect size of CdM vs. MSC on pulmonary vasculogenesis. Forest plots demonstrate SMD with 95% confidence interval.

**Additional file 6: Figure S6.** Effect size of CdM vs. MSC on lung permeability. Forest plots demonstrate SMD with 95% confidence interval.

**Additional file 7: Figure S7.** Effect size of CdM vs. MSC on pulmonary pressures. Forest plots demonstrate SMD with 95% confidence interval.

**Additional file 8: Figure S8.** Effect size of CdM (a), MSCs (b), and CdM vs. MSC (c) on all eight outcomes. Forest plots demonstrate SMD with 95% confidence interval.

**Additional file 9: Figure S9.** Effect size of CdM on lung alveolarization by disease (a), source (b), dose (c), and route (d). Forest plots demonstrate SMD with 95% confidence interval.

**Additional file 10: Figure S10.** Effect size of CdM on right ventricular hypertrophy by disease (a), source (b), dose (c), and route (d). Forest plots demonstrate SMD with 95% confidence interval.

**Additional file 11: Figure S11.** Effect size of CdM on lung fibrosis by disease (a), source (b), dose (c), and route (d). Forest plots demonstrate SMD with 95% confidence interval.

**Additional file 12: Figure S12.** Effect size of CdM on pulmonary vascularization by disease (a), source (b), dose (c), and route (d). Forest plots demonstrate SMD with 95% confidence interval.

**Additional file 13: Figure S13.** Funnel plot assessing for publication bias of CdM on lung alveolarization.

**Additional file 14: Figure S14.** Funnel plot assessing for publication bias of CdM on right ventricular hypertrophy.

**Additional file 15: Figure S15.** Funnel plot assessing for publication bias of CdM on lung fibrosis.

**Additional file 16: Figure S16.** Funnel plot assessing for publication bias of CdM on pulmonary vasculogenesis.

**Additional file 17: Figure S17.** Funnel plot assessing for publication bias of CdM on lung permeability.

**Additional file 18: Figure S18.** Funnel plot assessing for publication bias of CdM on pulmonary pressures.

**Additional file 19: Figure S19.** Funnel plot assessing for publication bias of CdM on histologic lung injury.

**Additional file 20: Figure S20.** Funnel plot assessing for publication bias of CdM on lung compliance.

**Additional file 21: File S1.** List of articles included in this review.

**Additional file 22: File S2.** SYRCLE risk of bias.

**Additional file 23: File S3.** CdM characteristics.

## Data Availability

Availability of data and materials will be available through figshare upon publication of the manuscripts.

## Abbreviations

ARDS: Acute respiratory distress syndrome
BPD: Bronchopulmonary dysplasia
CAMARADES: Collaborative Approach to Meta-Analysis and Review of Data from Experimental Studies
CdM: Conditioned media
CF: Cystic fibrosis
CI: Confidence interval
MSC: Mesenchymal stem/stromal cell
NCT: National clinical trial
PH: Pulmonary hypertension
SMD: Standardized mean difference
SYRCLE: Systematic Review Centre for Laboratory Animal Experimentation
