# Supplementary figures and images for: Effects of mesenchymal stromal cell-conditioned media on measures of lung structure and function: a systematic review and meta-analysis of preclinical studies
Source: Stem Cell Res Ther. 2020 Sep 15;11:399. doi: 10.1186/s13287-020-01900-7 (PMC7493362; doi:10.1186/s13287-020-01900-7)

Supplementary  
Figure 1

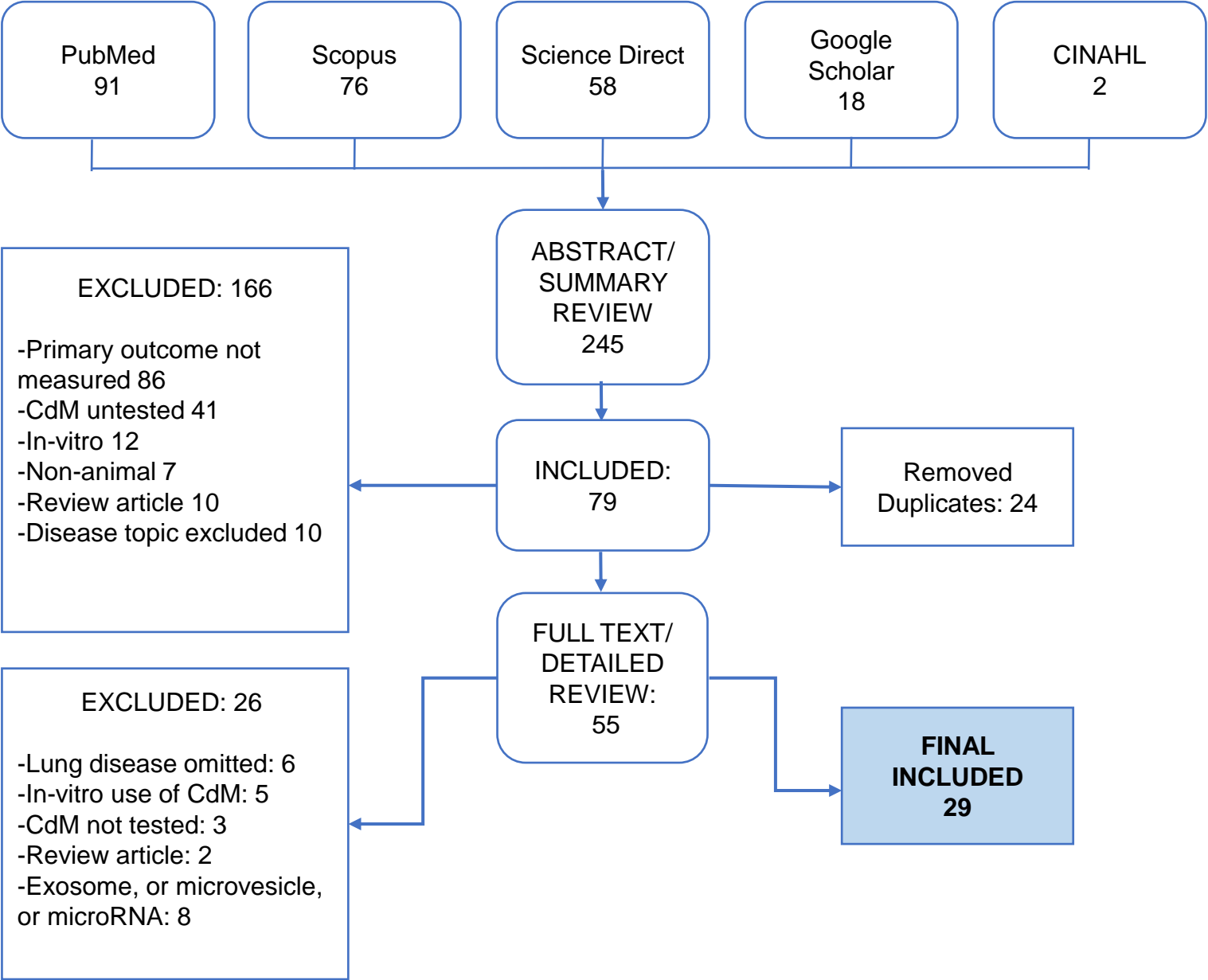

Supplement: Supplementary file 1 — Additional file 1: Figure S1. Flow diagram demonstrating study selection process. [file 13287_2020_1900_MOESM1_ESM.pdf]

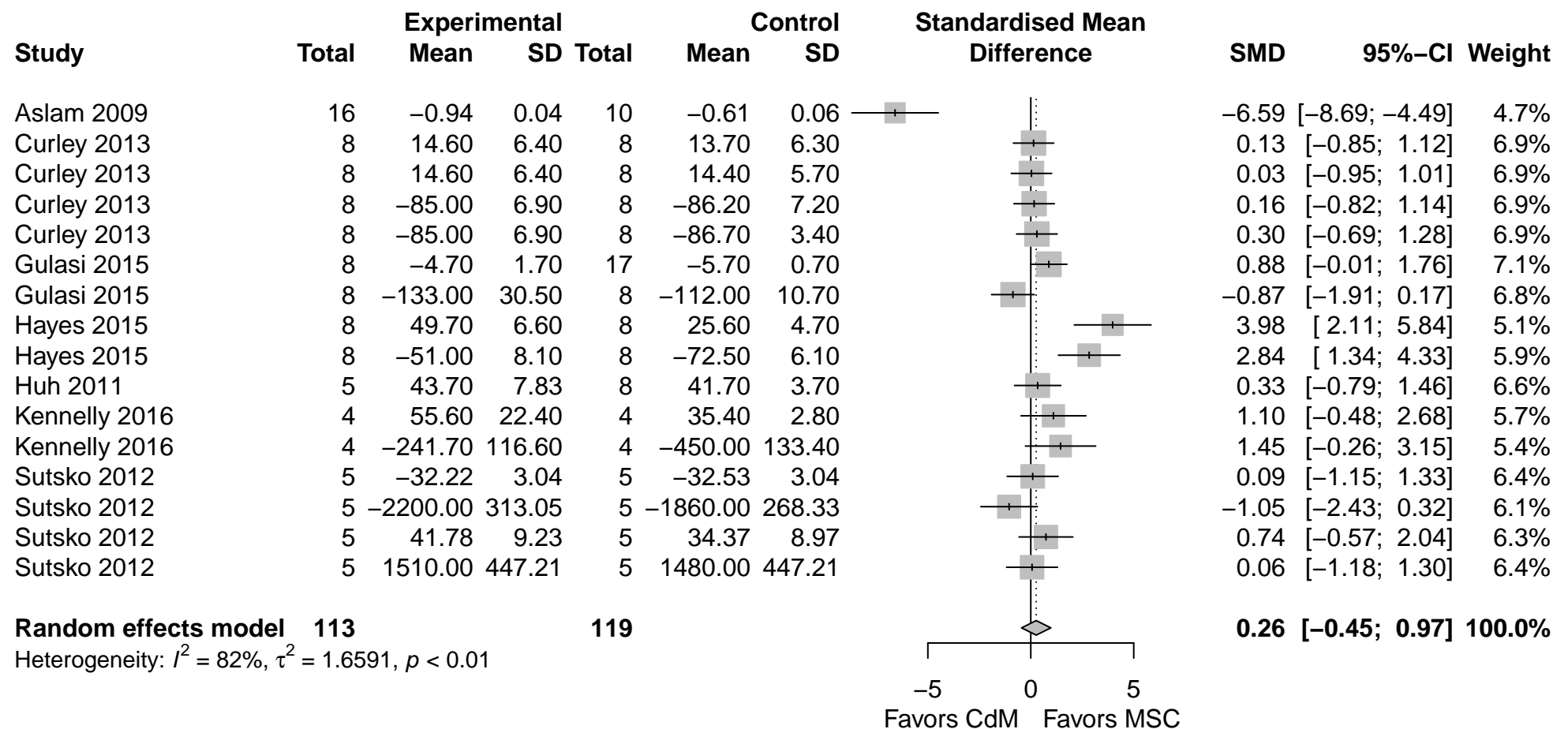

Supplement: Supplementary file 2 — Additional file 2: Figure S2. Effect size of CdM vs. MSC on lung alveolarization. . Forest plots demonstrate SMD with 95% confidence interval. [file 13287_2020_1900_MOESM2_ESM.pdf]

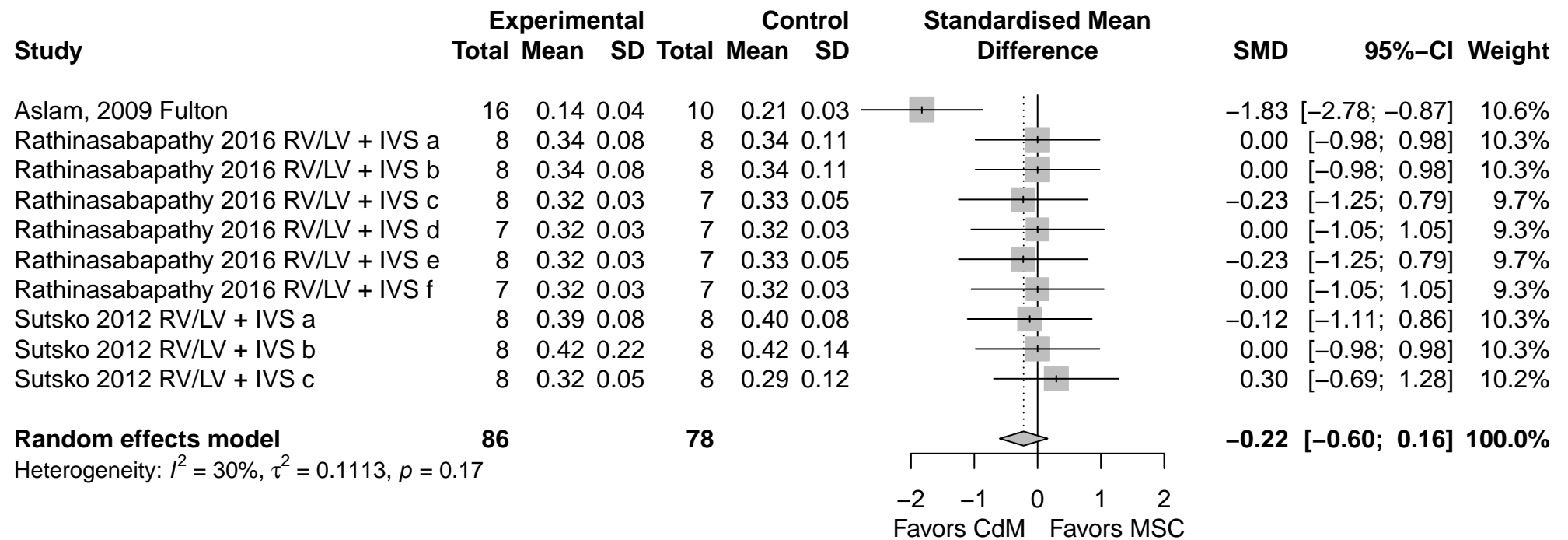

Supplement: Supplementary file 3 — Additional file 3: Figure S3. Effect size of CdM on right ventricular hypertrophy. Forest plots demonstrate SMD with 95% confidence interval. [file 13287_2020_1900_MOESM3_ESM.pdf]

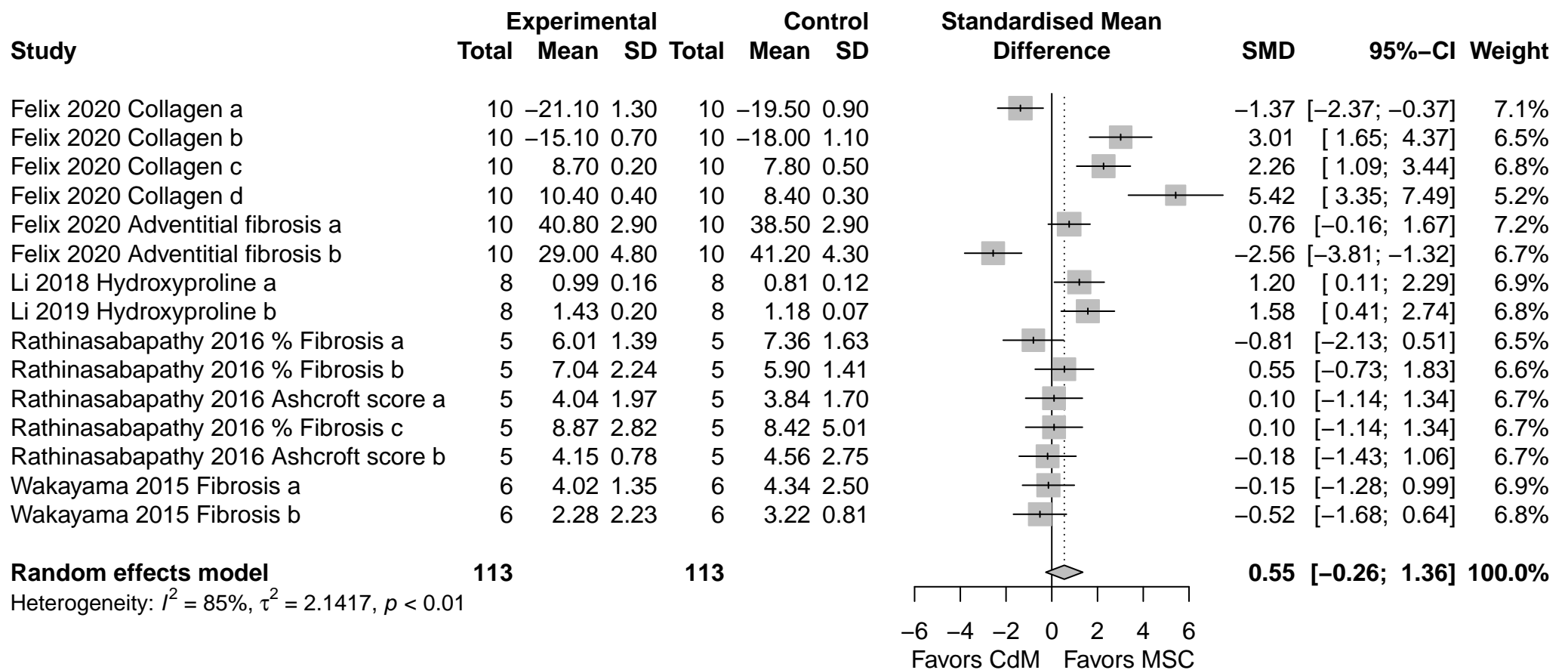

Supplement: Supplementary file 4 — Additional file 4: Figure S4. Effect size of MSC on lung fibrosis. Forest plots demonstrate SMD with 95% confidence interval. [file 13287_2020_1900_MOESM4_ESM.pdf]

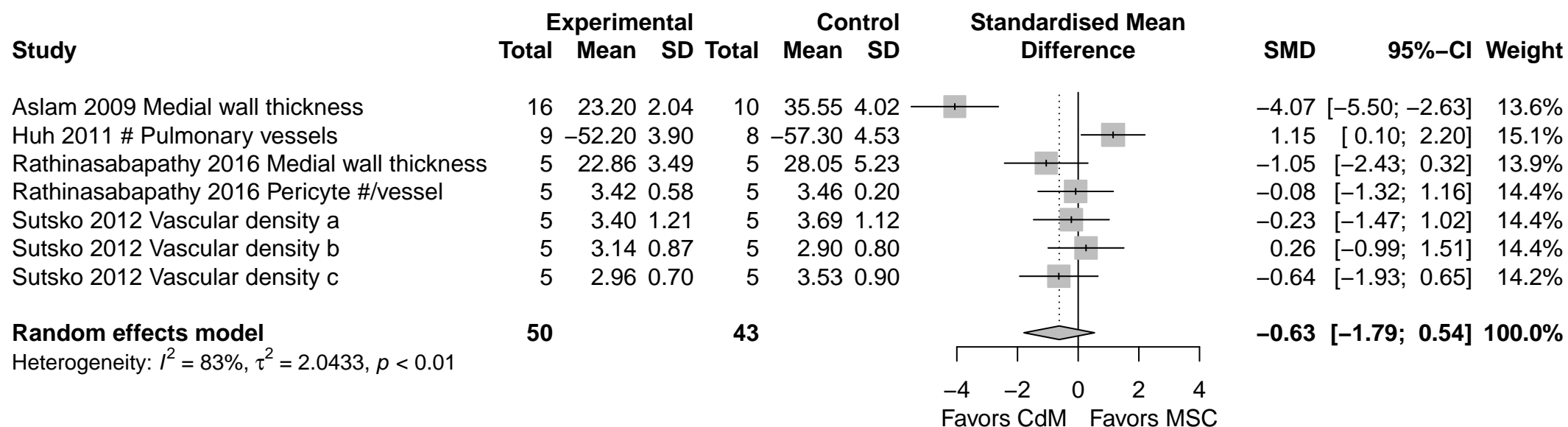

Supplement: Supplementary file 5 — Additional file 5: Figure S5. Effect size of CdM vs. MSC on pulmonary vasculogenesis. Forest plots demonstrate SMD with 95% confidence interval. [file 13287_2020_1900_MOESM5_ESM.pdf]

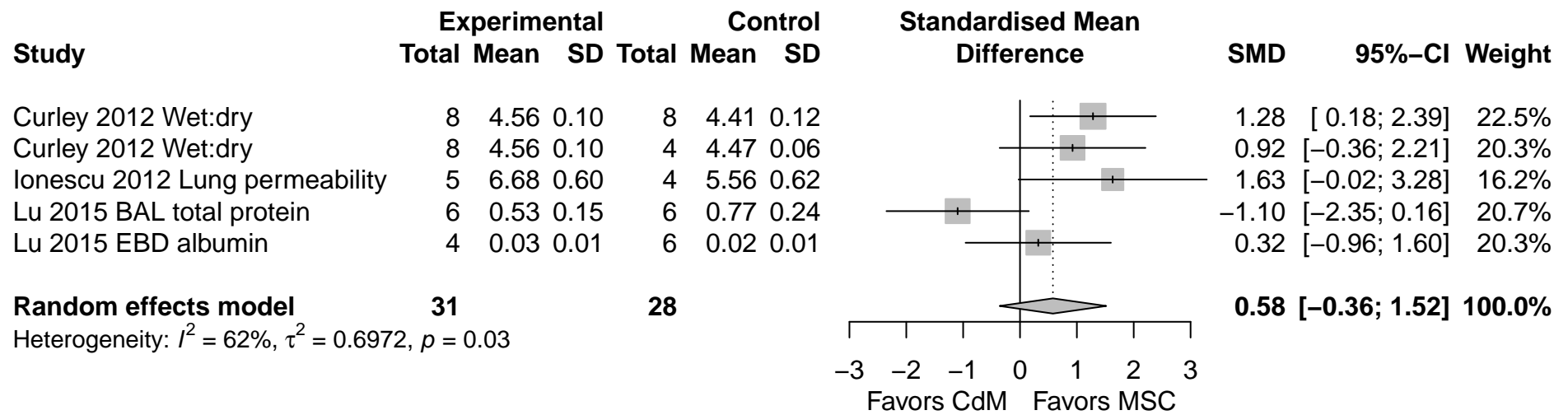

Supplement: Supplementary file 6 — Additional file 6: Figure S6. Effect size of CdM vs. MSC on lung permeability. Forest plots demonstrate SMD with 95% confidence interval. [file 13287_2020_1900_MOESM6_ESM.pdf]

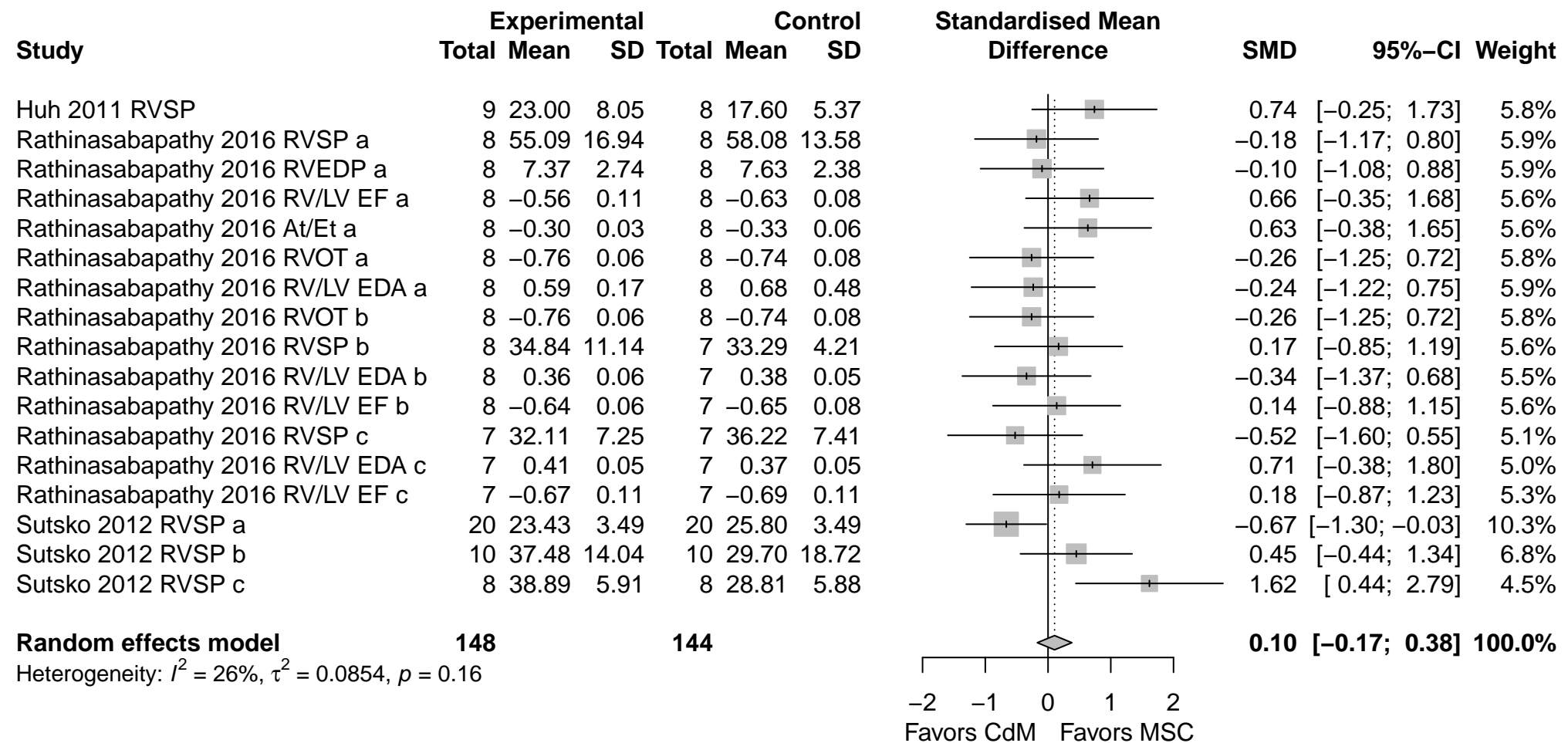

Supplement: Supplementary file 7 — Additional file 7: Figure S7. Effect size of CdM vs. MSC on pulmonary pressures. Forest plots demonstrate SMD with 95% confidence interval. [file 13287_2020_1900_MOESM7_ESM.pdf]

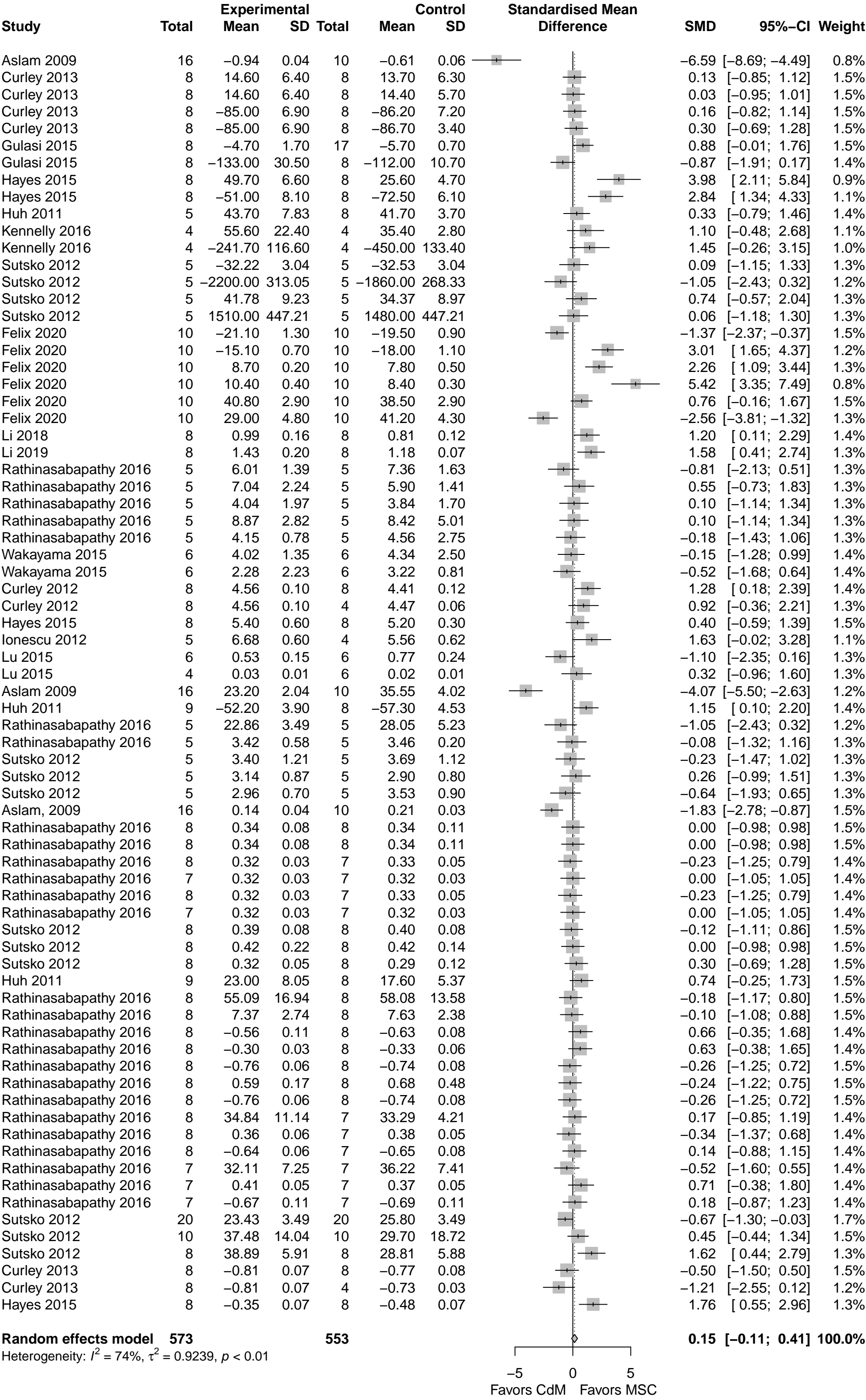

Supplement: Supplementary file 8 — Additional file 8: Figure S8. Effect size of CdM (a), MSCs (b), and CdM vs. MSC (c) on all eight outcomes. Forest plots demonstrate SMD with 95% confidence interval. [file 13287_2020_1900_MOESM8_ESM.zip › OVERALL_CdM vs MSC.pdf]

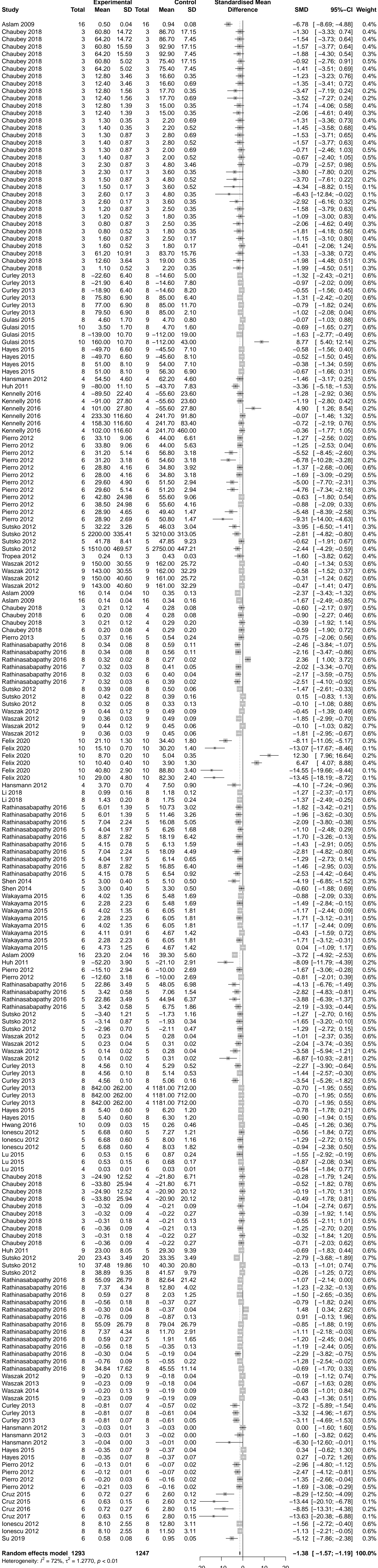

Supplement: Supplementary file 8 — Additional file 8: Figure S8. Effect size of CdM (a), MSCs (b), and CdM vs. MSC (c) on all eight outcomes. Forest plots demonstrate SMD with 95% confidence interval. [file 13287_2020_1900_MOESM8_ESM.zip › OVERALL_CdM.pdf]

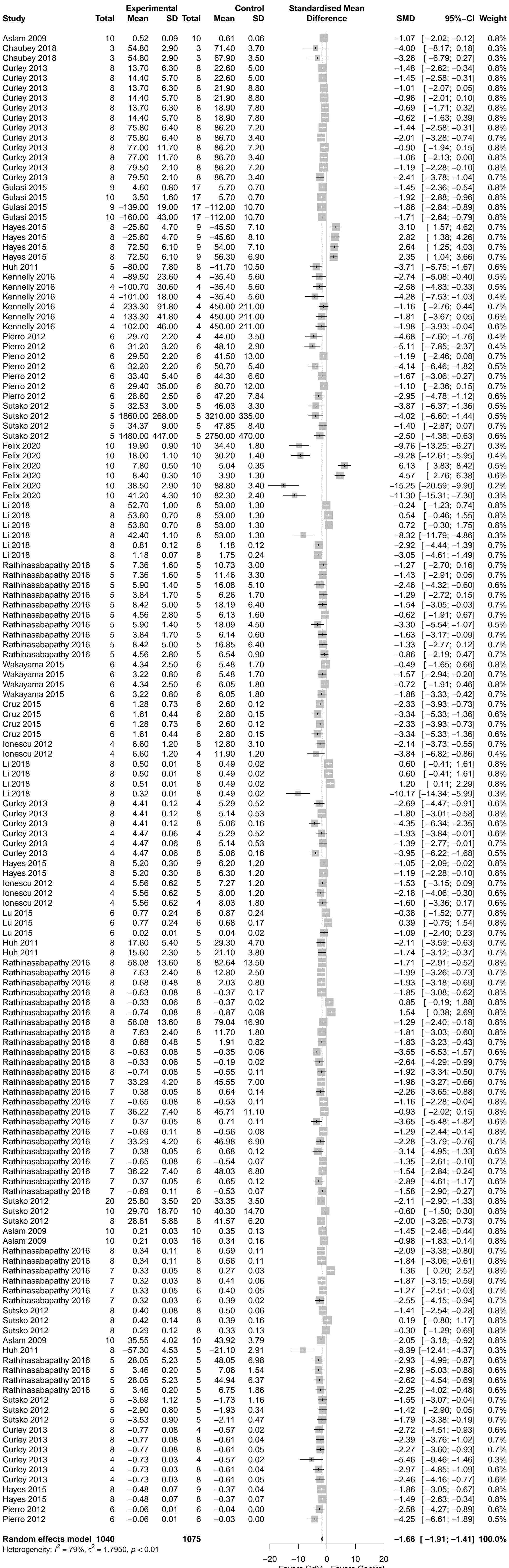

Supplement: Supplementary file 8 — Additional file 8: Figure S8. Effect size of CdM (a), MSCs (b), and CdM vs. MSC (c) on all eight outcomes. Forest plots demonstrate SMD with 95% confidence interval. [file 13287_2020_1900_MOESM8_ESM.zip › OVERALL_MSC.pdf]

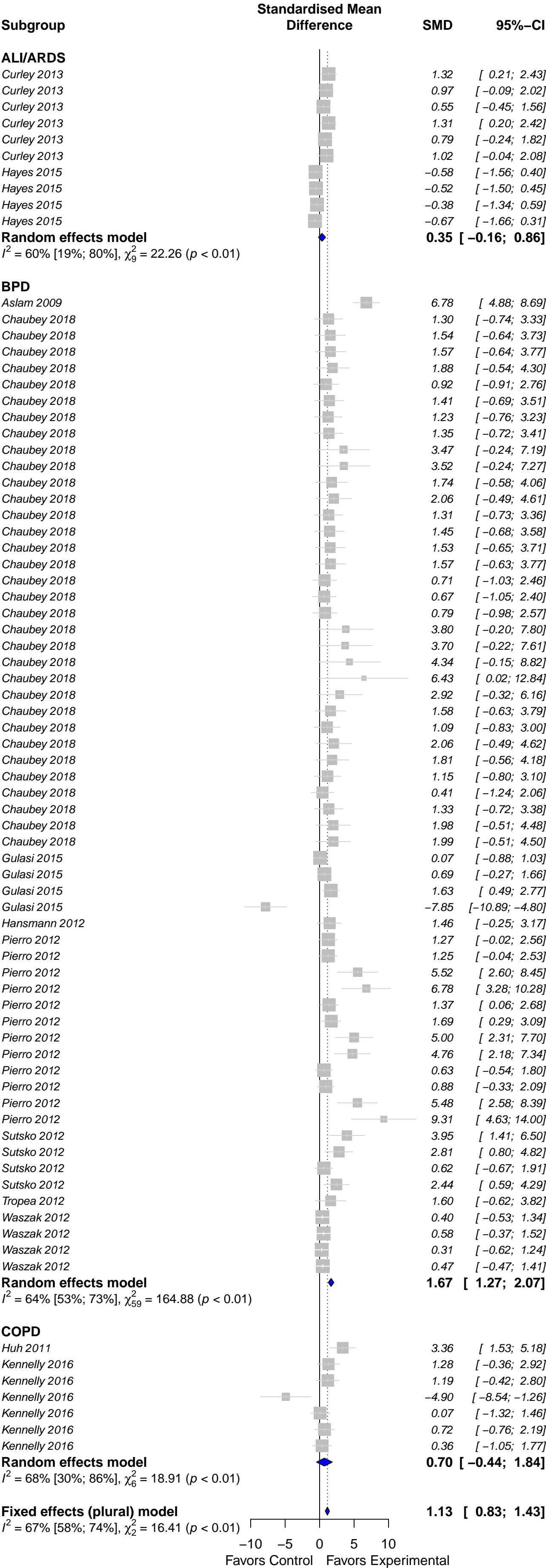

Supplement: Supplementary file 9 — Additional file 9: Figure S9. Effect size of CdM on lung alveolarization by disease (a), source (b), dose (c), and route (d). Forest plots demonstrate SMD with 95% confidence interval. [file 13287_2020_1900_MOESM9_ESM.zip › S. Fig 9A. Alv_disease.pdf]

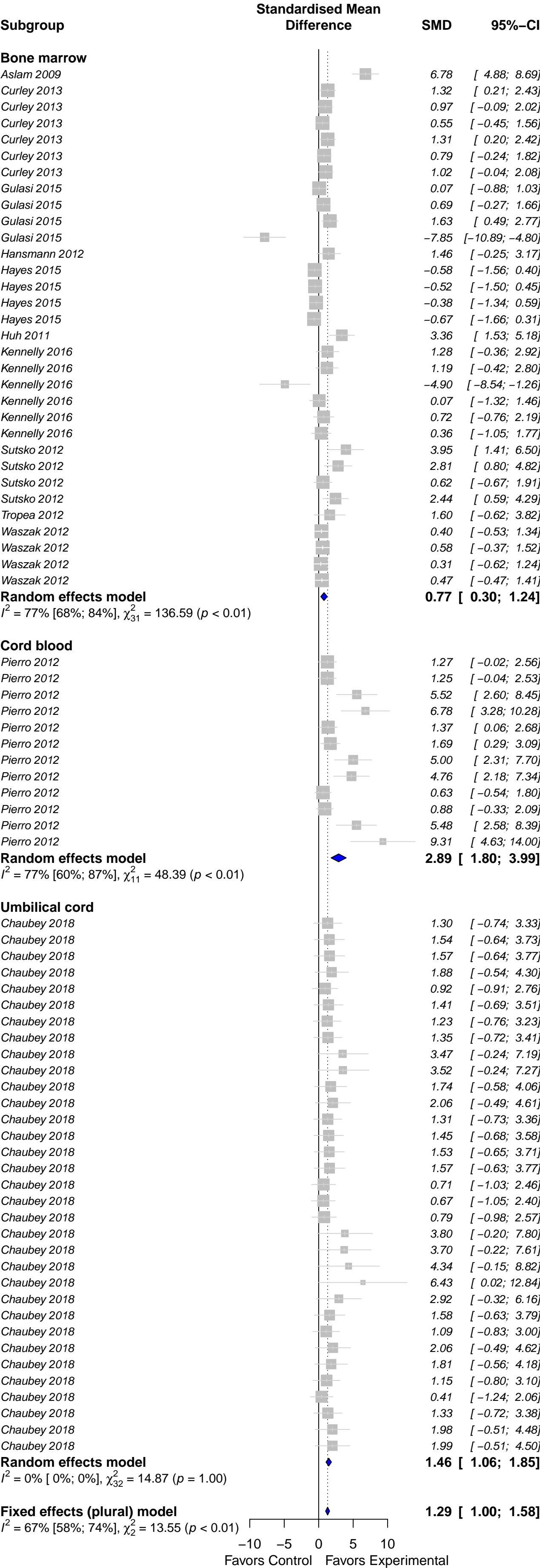

-10 -5 0 5 10  
Favors Control Favors Experimental

Supplement: Supplementary file 9 — Additional file 9: Figure S9. Effect size of CdM on lung alveolarization by disease (a), source (b), dose (c), and route (d). Forest plots demonstrate SMD with 95% confidence interval. [file 13287_2020_1900_MOESM9_ESM.zip › S. Fig 9B. Alv source.pdf]

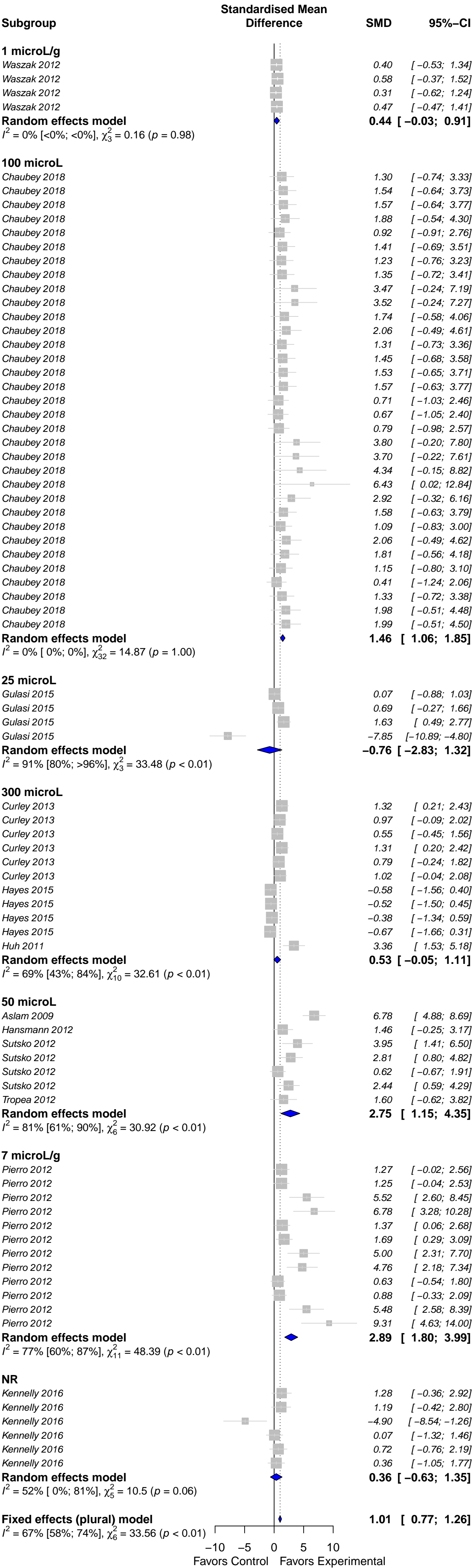

Supplement: Supplementary file 9 — Additional file 9: Figure S9. Effect size of CdM on lung alveolarization by disease (a), source (b), dose (c), and route (d). Forest plots demonstrate SMD with 95% confidence interval. [file 13287_2020_1900_MOESM9_ESM.zip › S. Fig 9C. Alv dose.pdf]

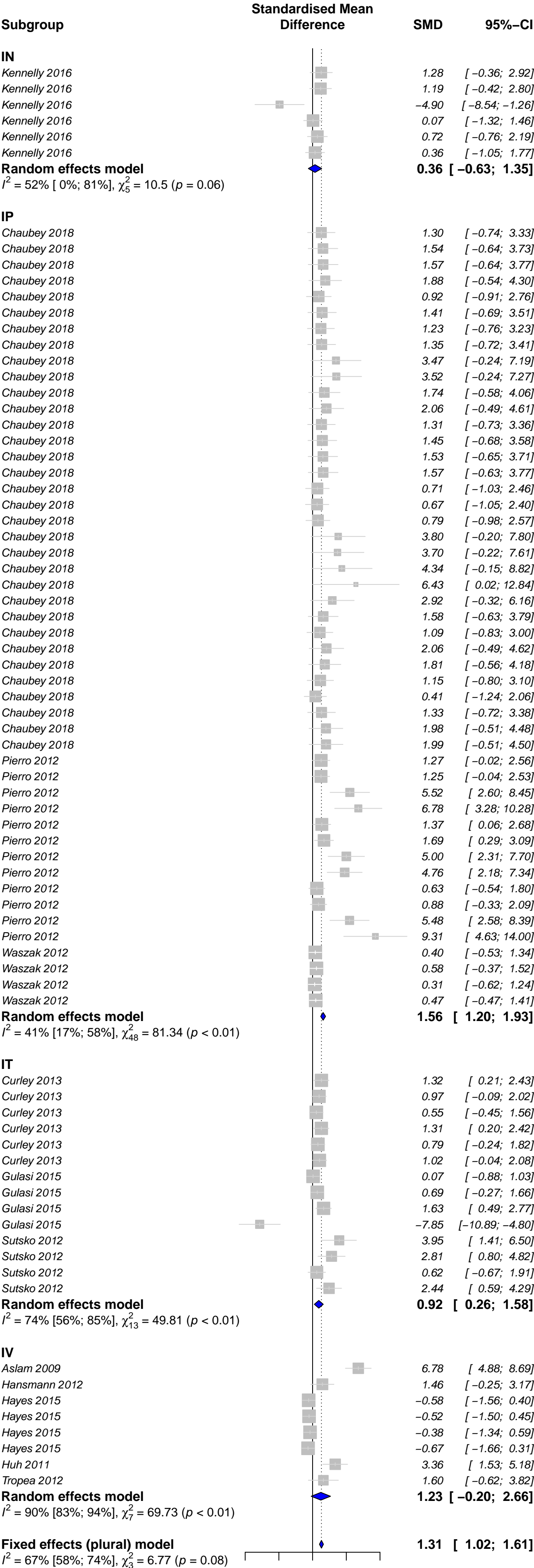

Supplement: Supplementary file 9 — Additional file 9: Figure S9. Effect size of CdM on lung alveolarization by disease (a), source (b), dose (c), and route (d). Forest plots demonstrate SMD with 95% confidence interval. [file 13287_2020_1900_MOESM9_ESM.zip › S. Fig 9D. Alv route.pdf]

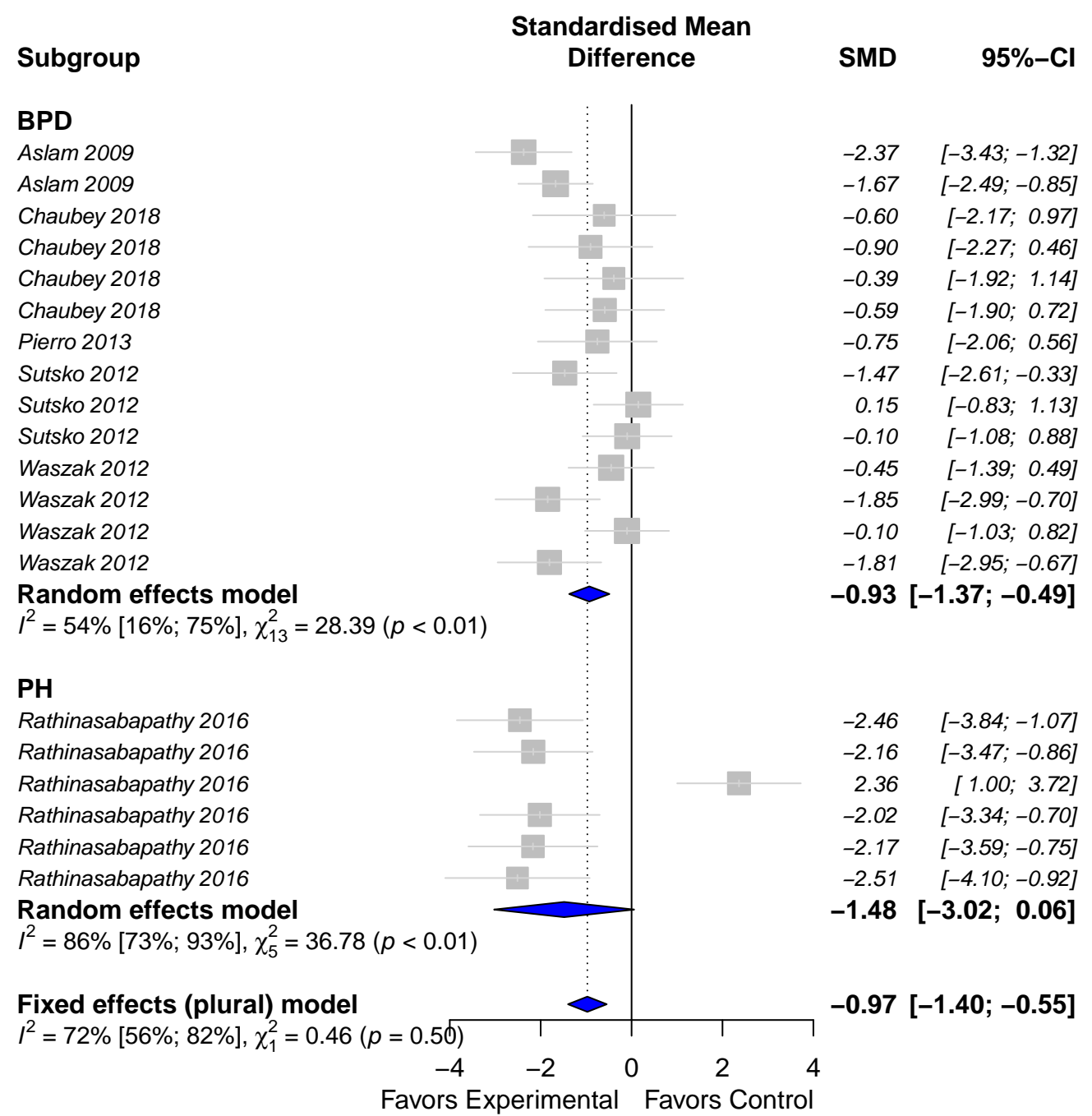

Supplement: Supplementary file 10 — Additional file 10: Figure S10. Effect size of CdM on right ventricular hypertrophy by disease (a), source (b), dose (c), and route (d). Forest plots demonstrate SMD with 95% confidence interval. [file 13287_2020_1900_MOESM10_ESM.zip › S10A.pdf]

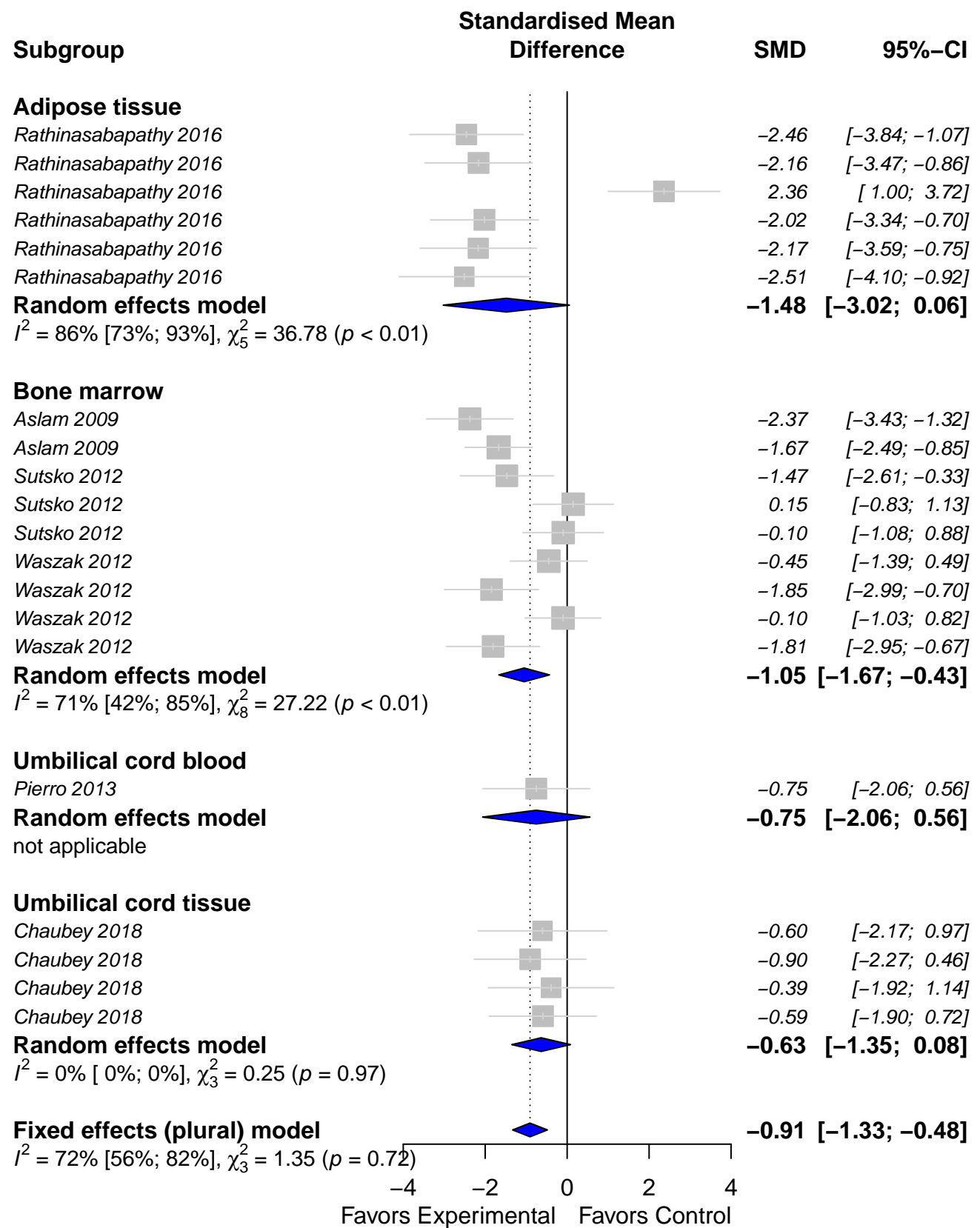

Supplement: Supplementary file 10 — Additional file 10: Figure S10. Effect size of CdM on right ventricular hypertrophy by disease (a), source (b), dose (c), and route (d). Forest plots demonstrate SMD with 95% confidence interval. [file 13287_2020_1900_MOESM10_ESM.zip › S10B.pdf]

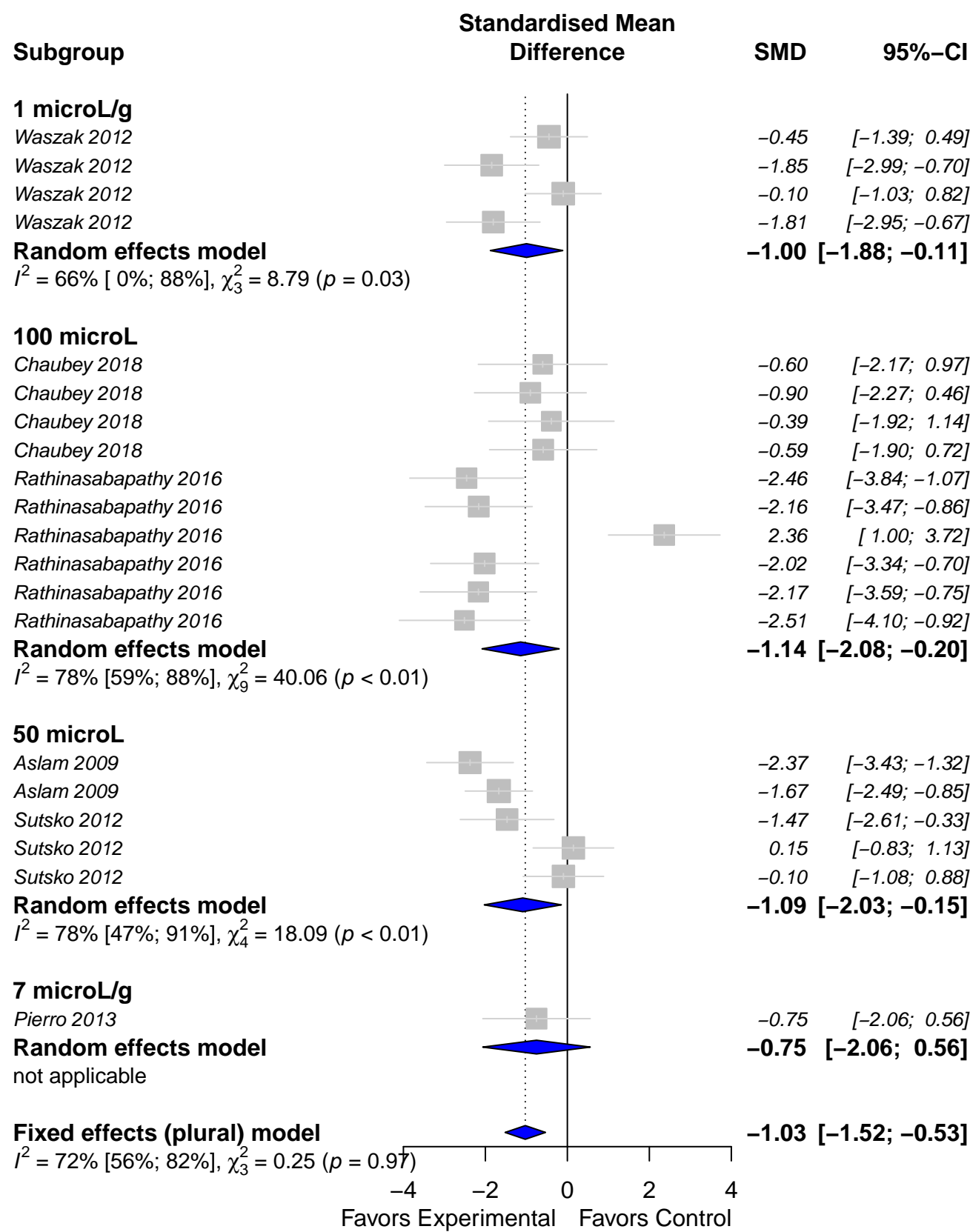

Supplement: Supplementary file 10 — Additional file 10: Figure S10. Effect size of CdM on right ventricular hypertrophy by disease (a), source (b), dose (c), and route (d). Forest plots demonstrate SMD with 95% confidence interval. [file 13287_2020_1900_MOESM10_ESM.zip › S10C.pdf]

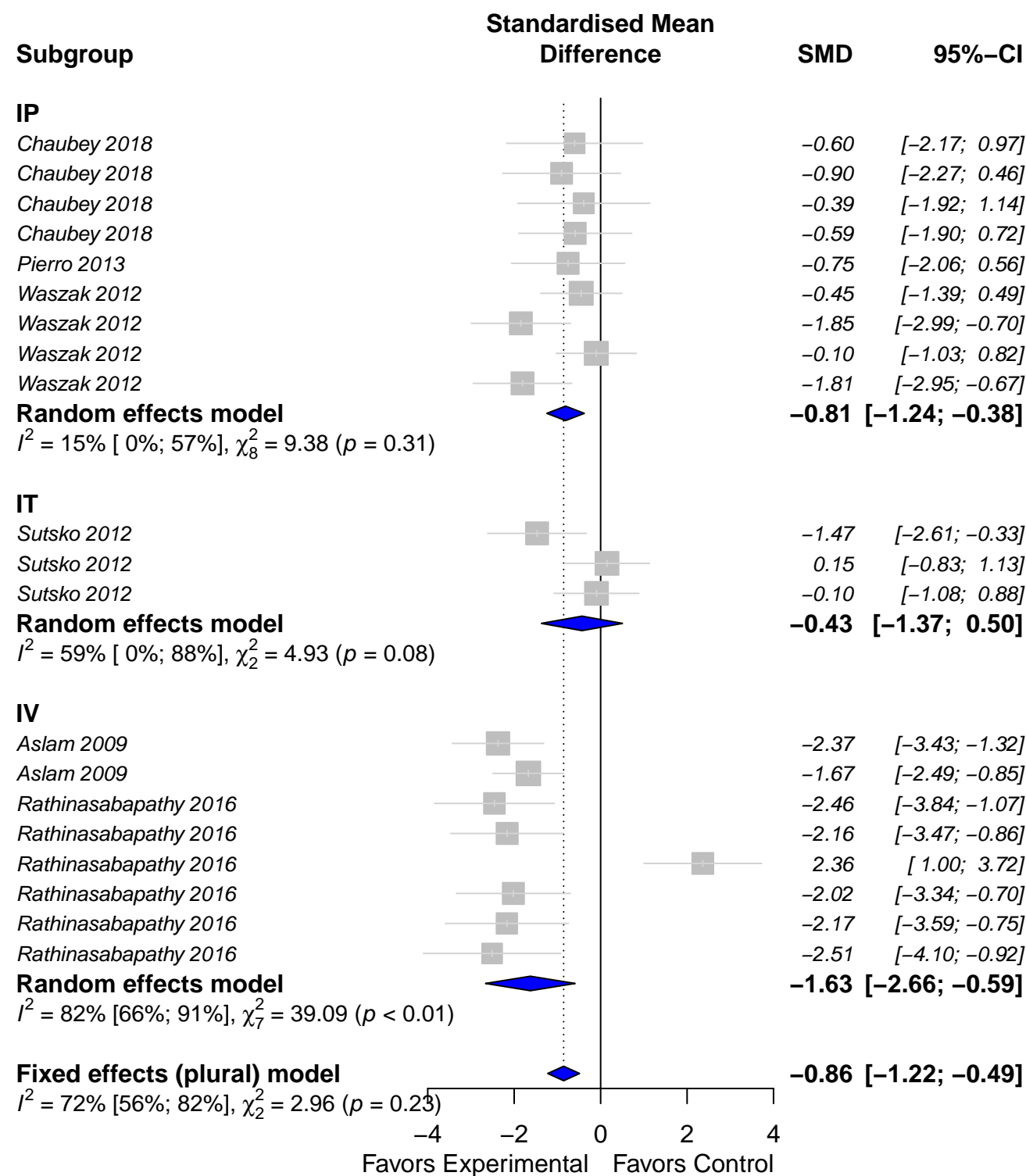

Supplement: Supplementary file 10 — Additional file 10: Figure S10. Effect size of CdM on right ventricular hypertrophy by disease (a), source (b), dose (c), and route (d). Forest plots demonstrate SMD with 95% confidence interval. [file 13287_2020_1900_MOESM10_ESM.zip › S10D.pdf]

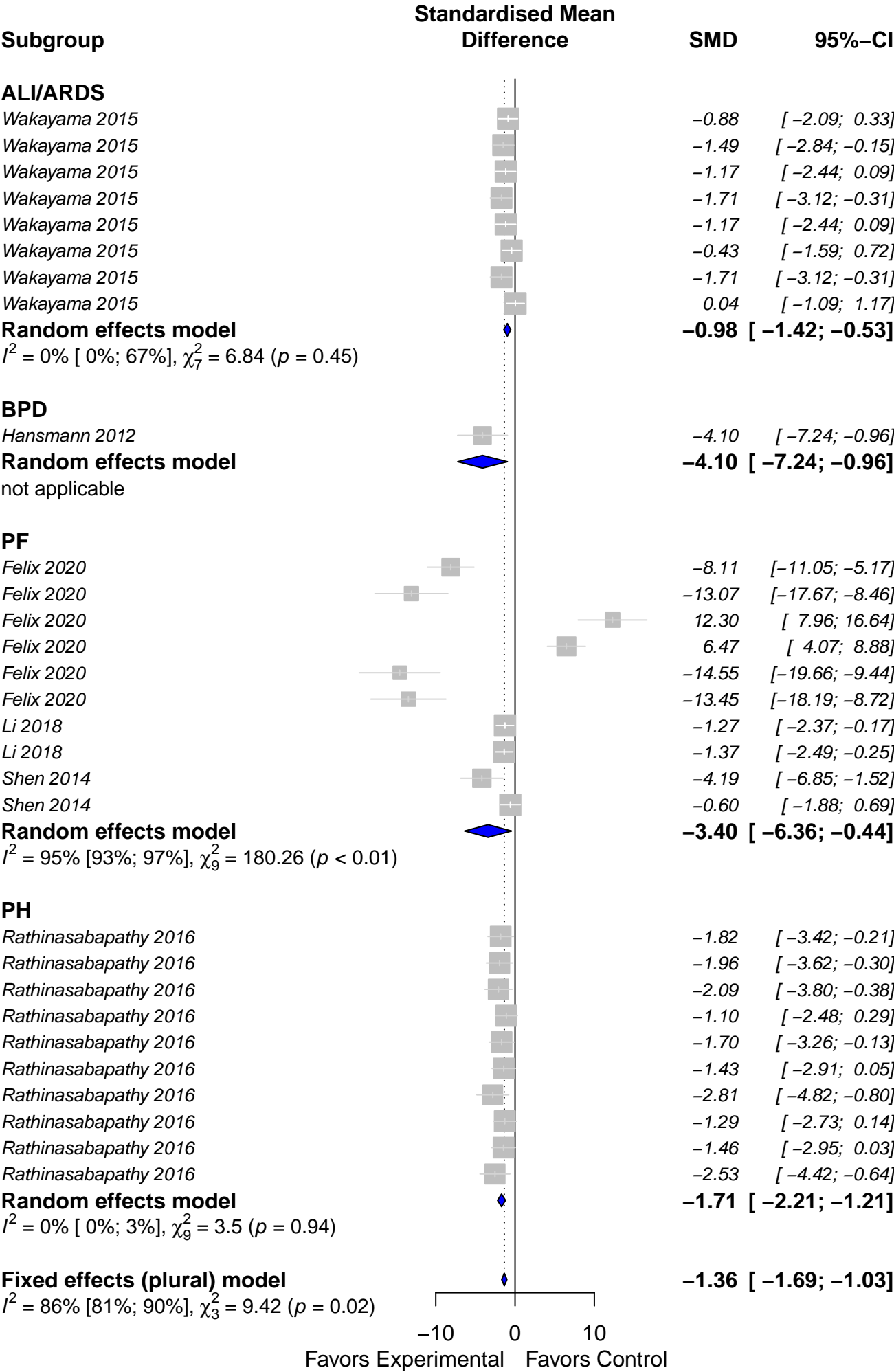

Supplement: Supplementary file 11 — Additional file 11: Figure S11. Effect size of CdM on lung fibrosis by disease (a), source (b), dose (c), and route (d). Forest plots demonstrate SMD with 95% confidence interval. [file 13287_2020_1900_MOESM11_ESM.zip › S11A.pdf]

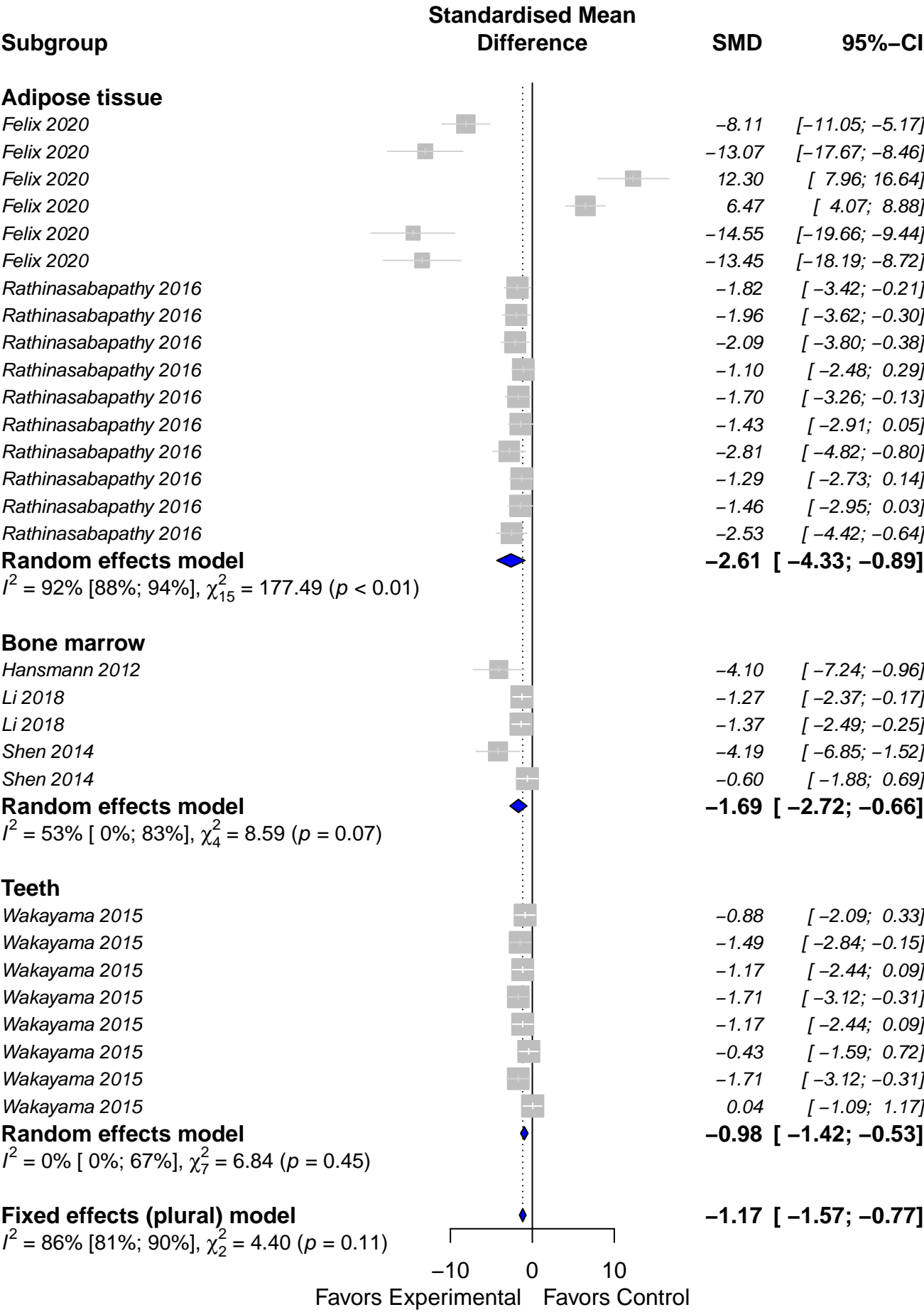

Supplement: Supplementary file 11 — Additional file 11: Figure S11. Effect size of CdM on lung fibrosis by disease (a), source (b), dose (c), and route (d). Forest plots demonstrate SMD with 95% confidence interval. [file 13287_2020_1900_MOESM11_ESM.zip › S11B.pdf]

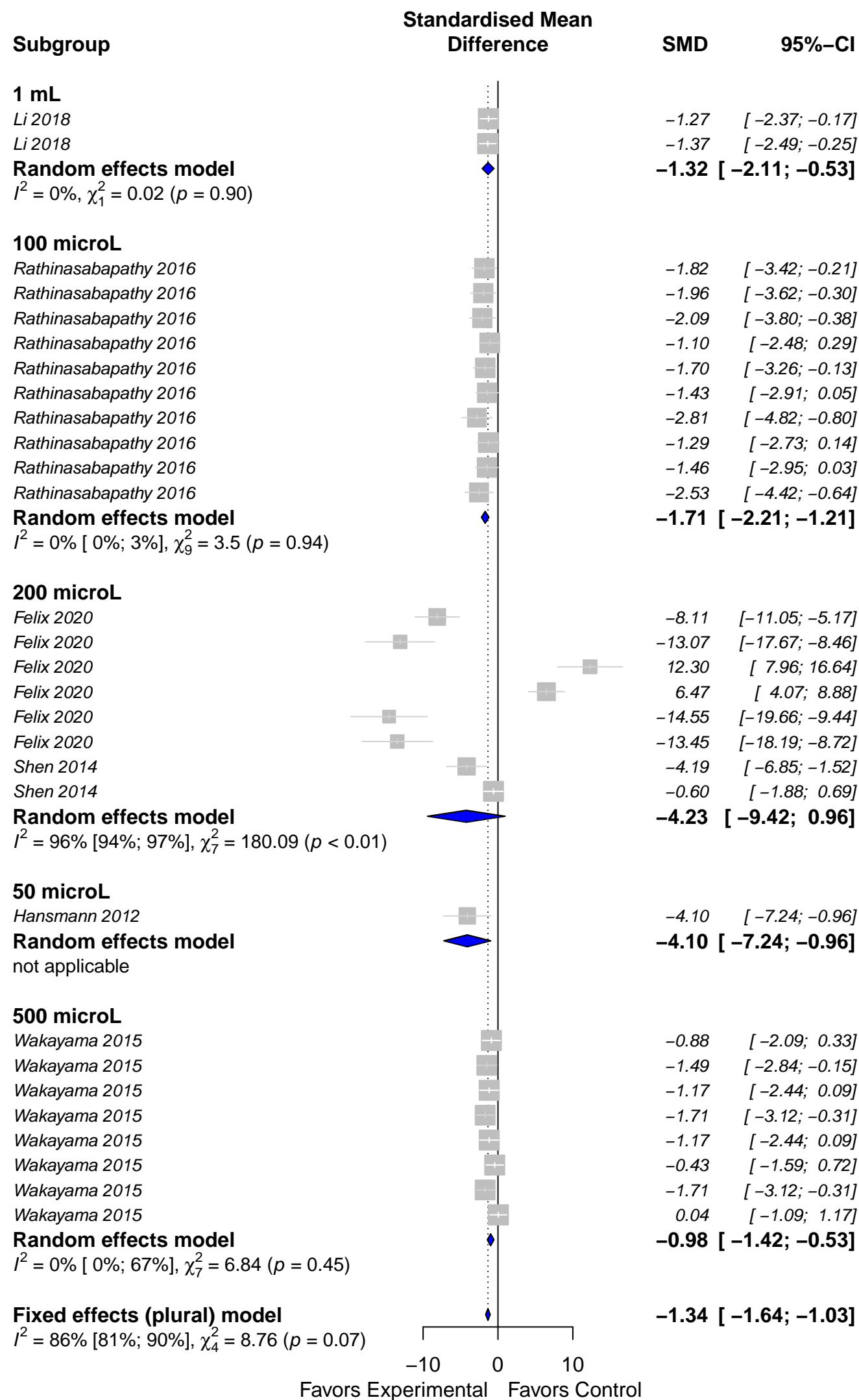

Supplement: Supplementary file 11 — Additional file 11: Figure S11. Effect size of CdM on lung fibrosis by disease (a), source (b), dose (c), and route (d). Forest plots demonstrate SMD with 95% confidence interval. [file 13287_2020_1900_MOESM11_ESM.zip › S11C.pdf]

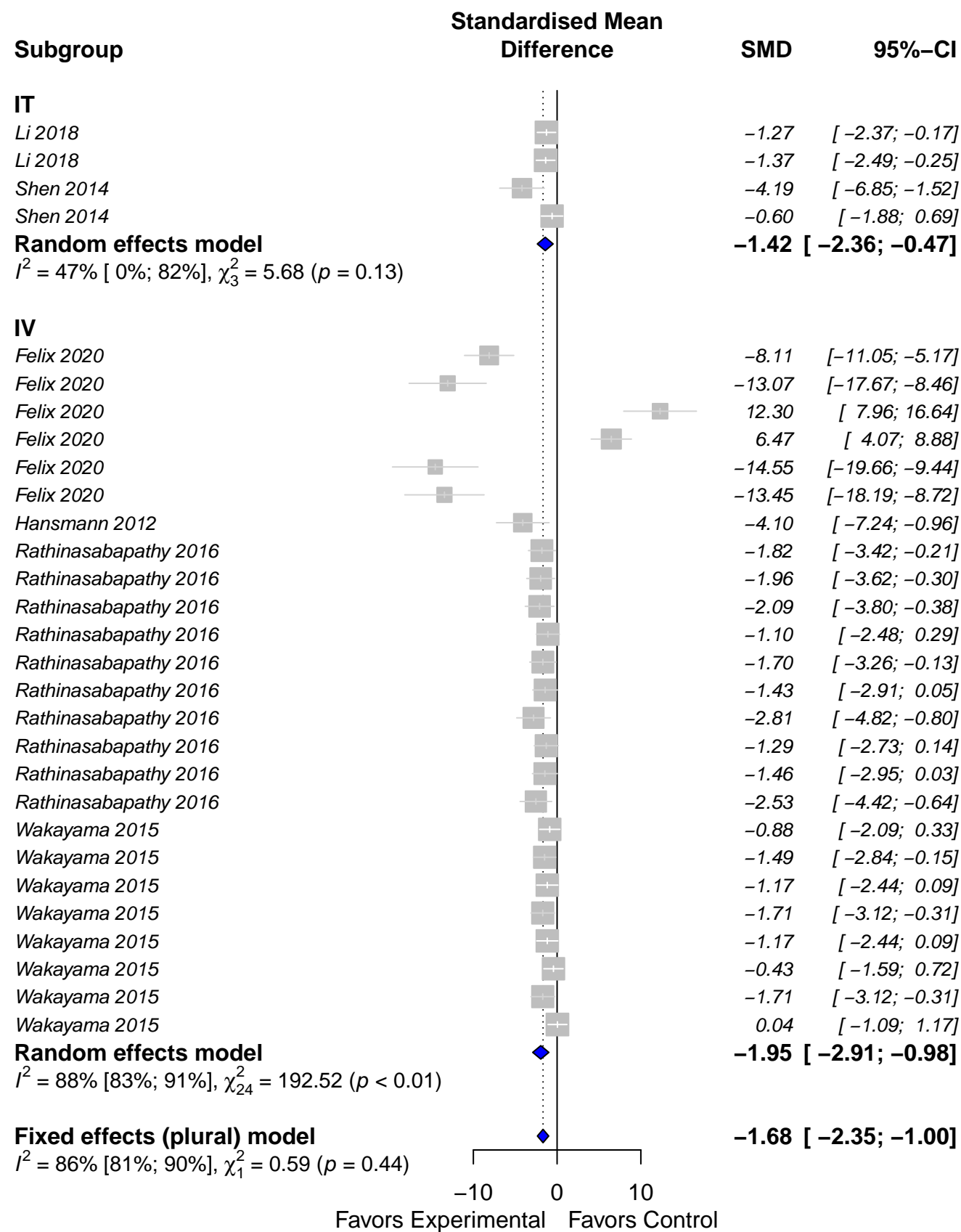

Supplement: Supplementary file 11 — Additional file 11: Figure S11. Effect size of CdM on lung fibrosis by disease (a), source (b), dose (c), and route (d). Forest plots demonstrate SMD with 95% confidence interval. [file 13287_2020_1900_MOESM11_ESM.zip › S11D.pdf]

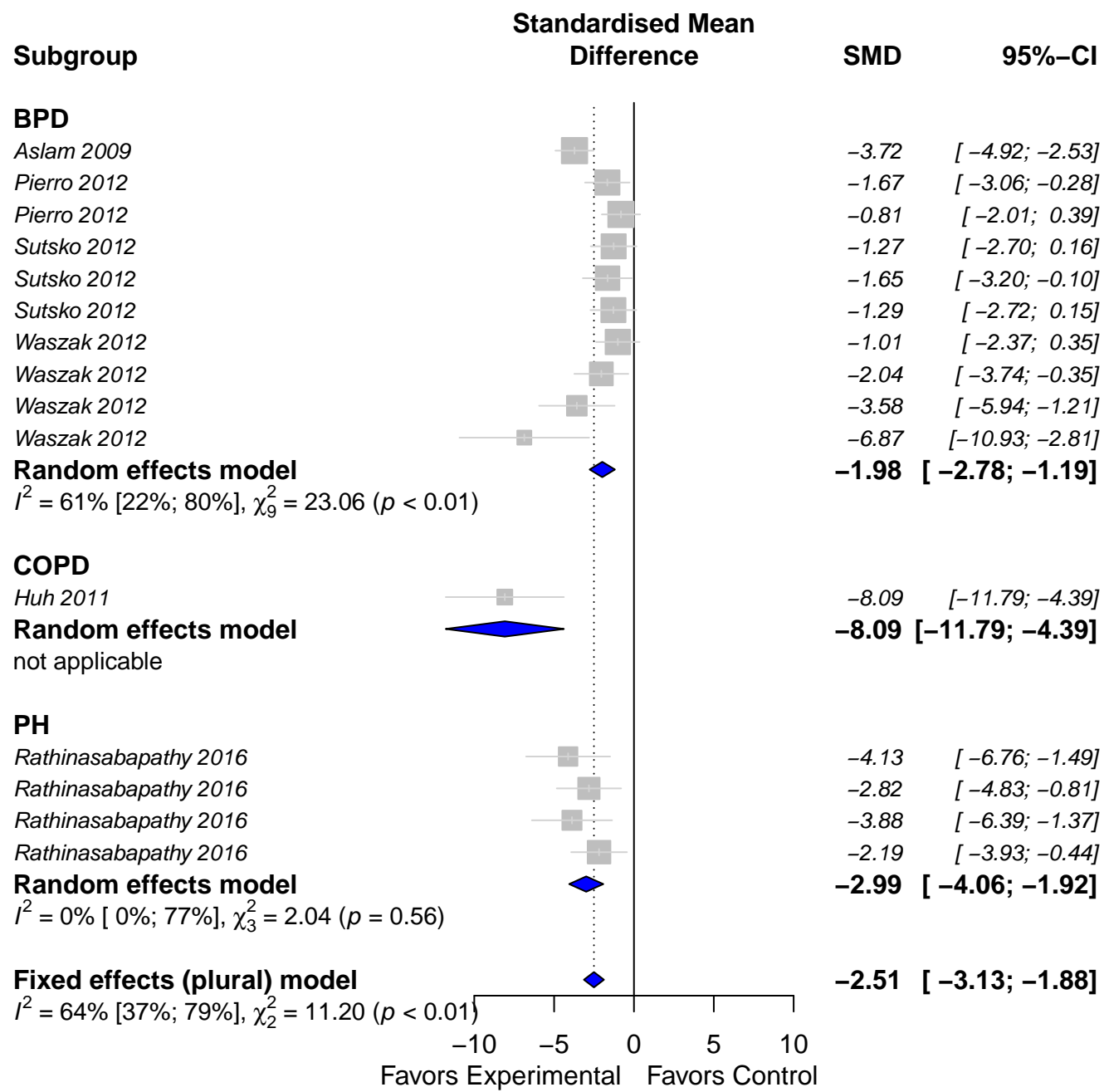

Supplement: Supplementary file 12 — Additional file 12: Figure S12. Effect size of CdM on pulmonary vascularization by disease (a), source (b), dose (c), and route (d). Forest plots demonstrate SMD with 95% confidence interval. [file 13287_2020_1900_MOESM12_ESM.zip › S12A.pdf]

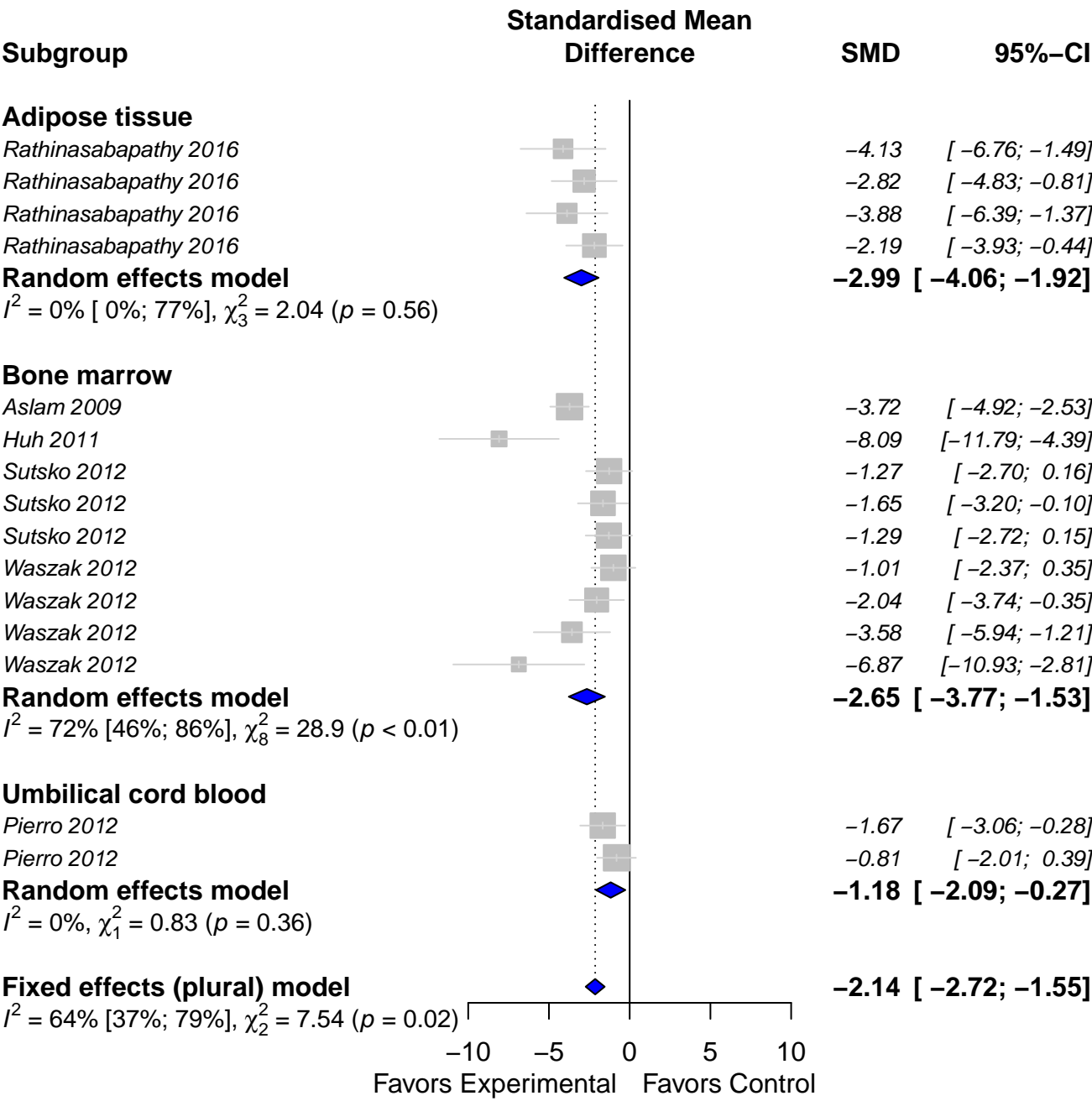

Supplement: Supplementary file 12 — Additional file 12: Figure S12. Effect size of CdM on pulmonary vascularization by disease (a), source (b), dose (c), and route (d). Forest plots demonstrate SMD with 95% confidence interval. [file 13287_2020_1900_MOESM12_ESM.zip › S12B.pdf]

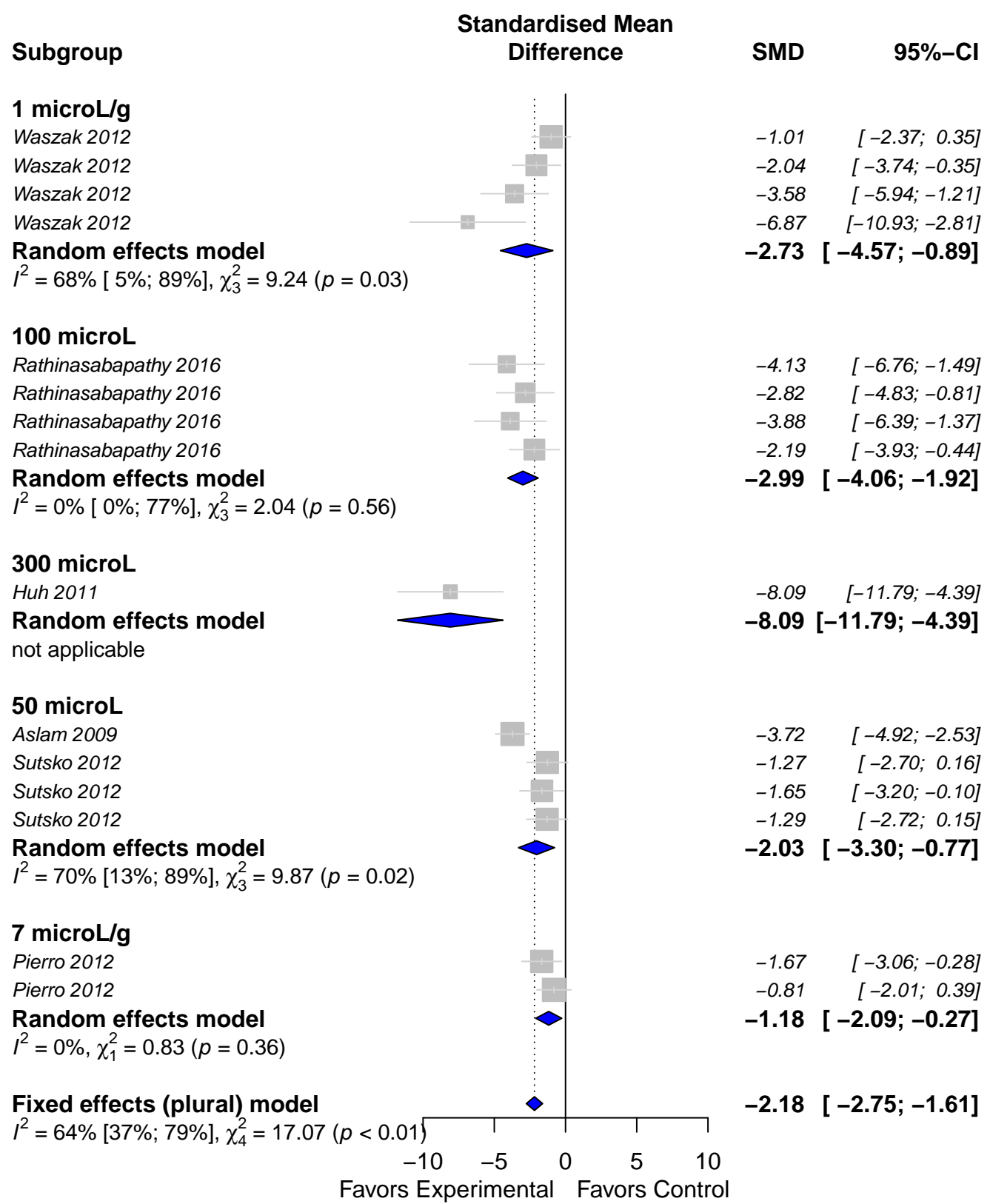

Supplement: Supplementary file 12 — Additional file 12: Figure S12. Effect size of CdM on pulmonary vascularization by disease (a), source (b), dose (c), and route (d). Forest plots demonstrate SMD with 95% confidence interval. [file 13287_2020_1900_MOESM12_ESM.zip › S12C.pdf]

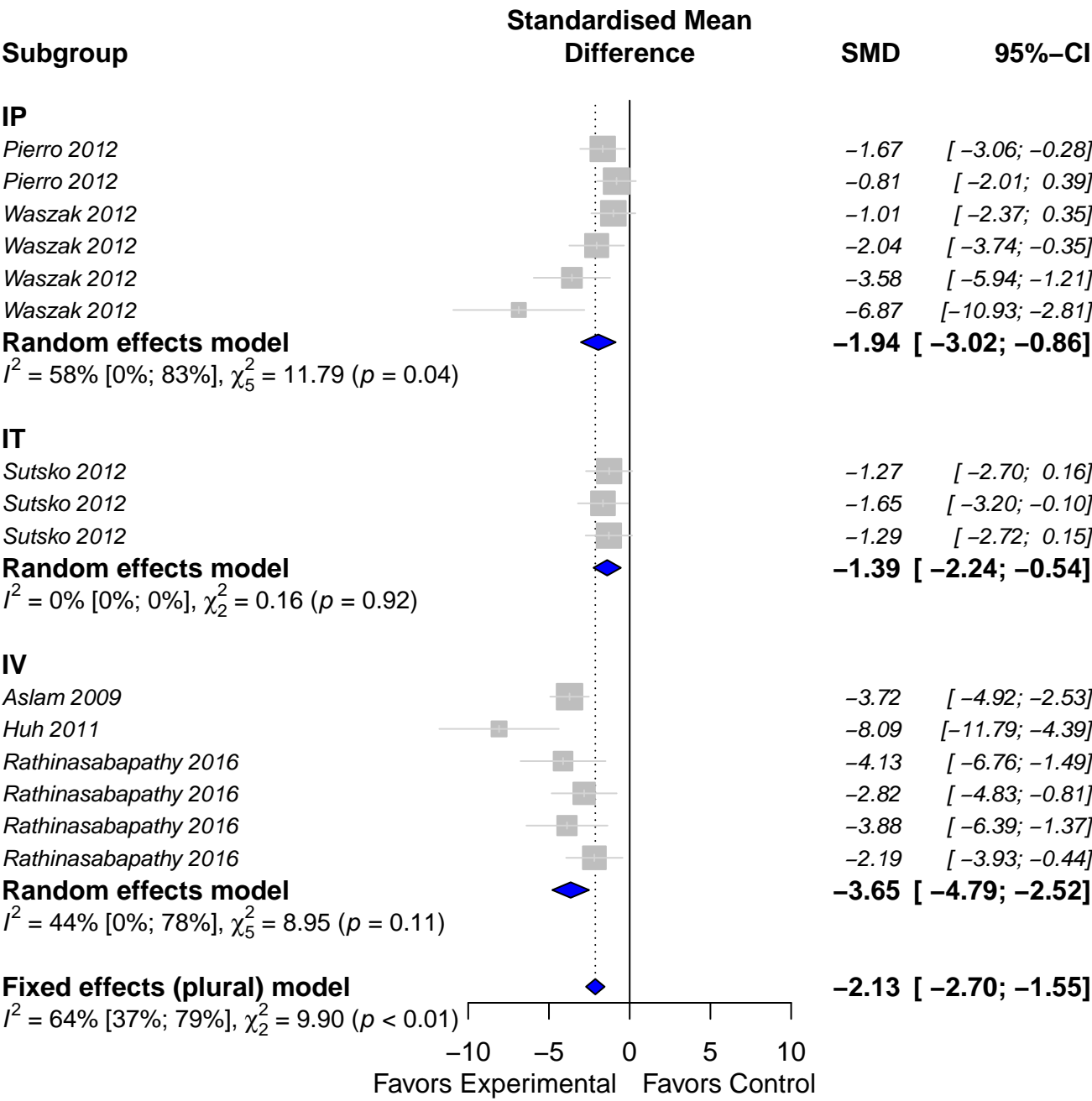

Supplement: Supplementary file 12 — Additional file 12: Figure S12. Effect size of CdM on pulmonary vascularization by disease (a), source (b), dose (c), and route (d). Forest plots demonstrate SMD with 95% confidence interval. [file 13287_2020_1900_MOESM12_ESM.zip › S12D.pdf]

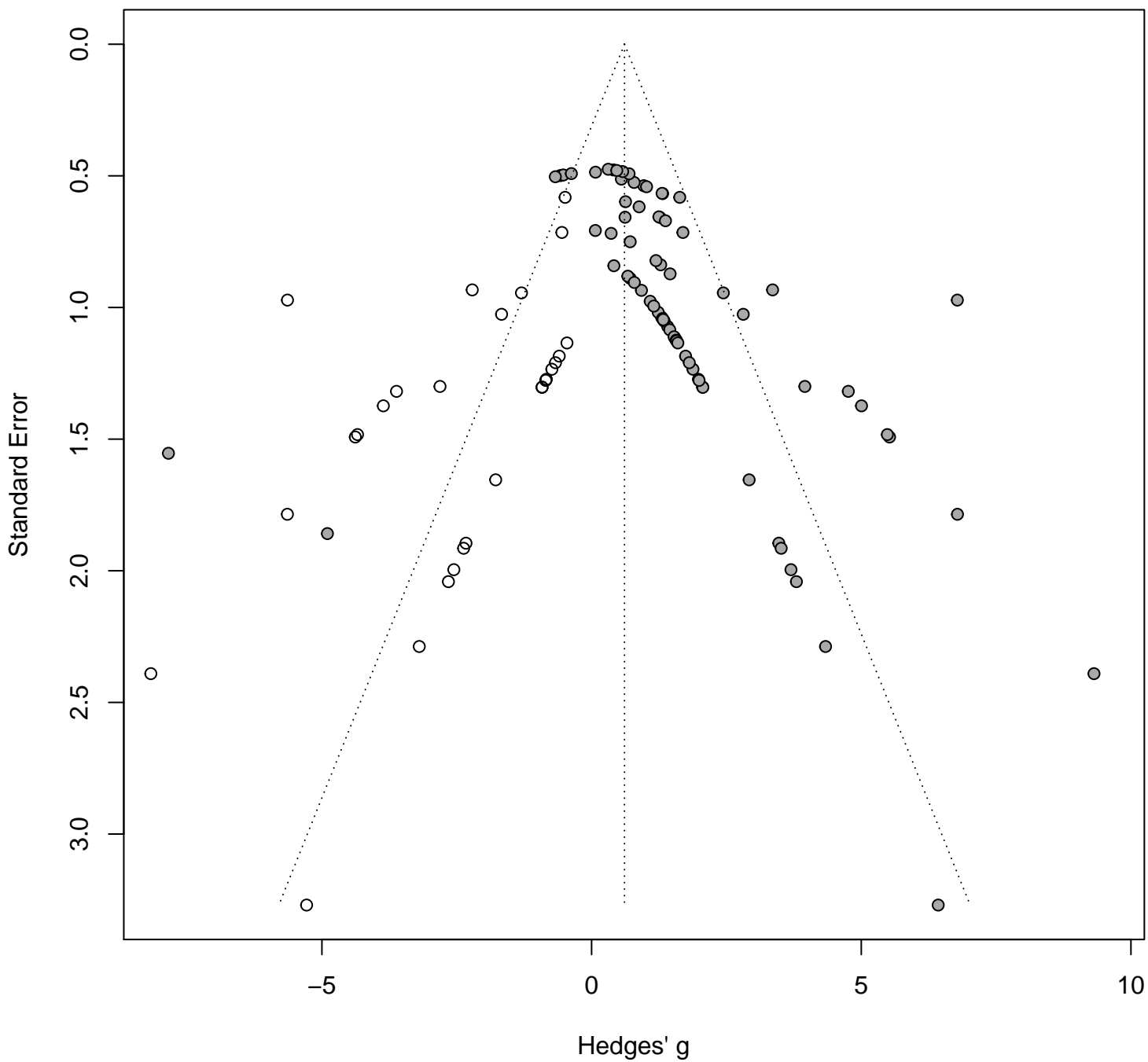

Supplement: Supplementary file 13 — Additional file 13: Figure S13. Funnel plot assessing for publication bias of CdM on lung alveolarization. [file 13287_2020_1900_MOESM13_ESM.pdf]

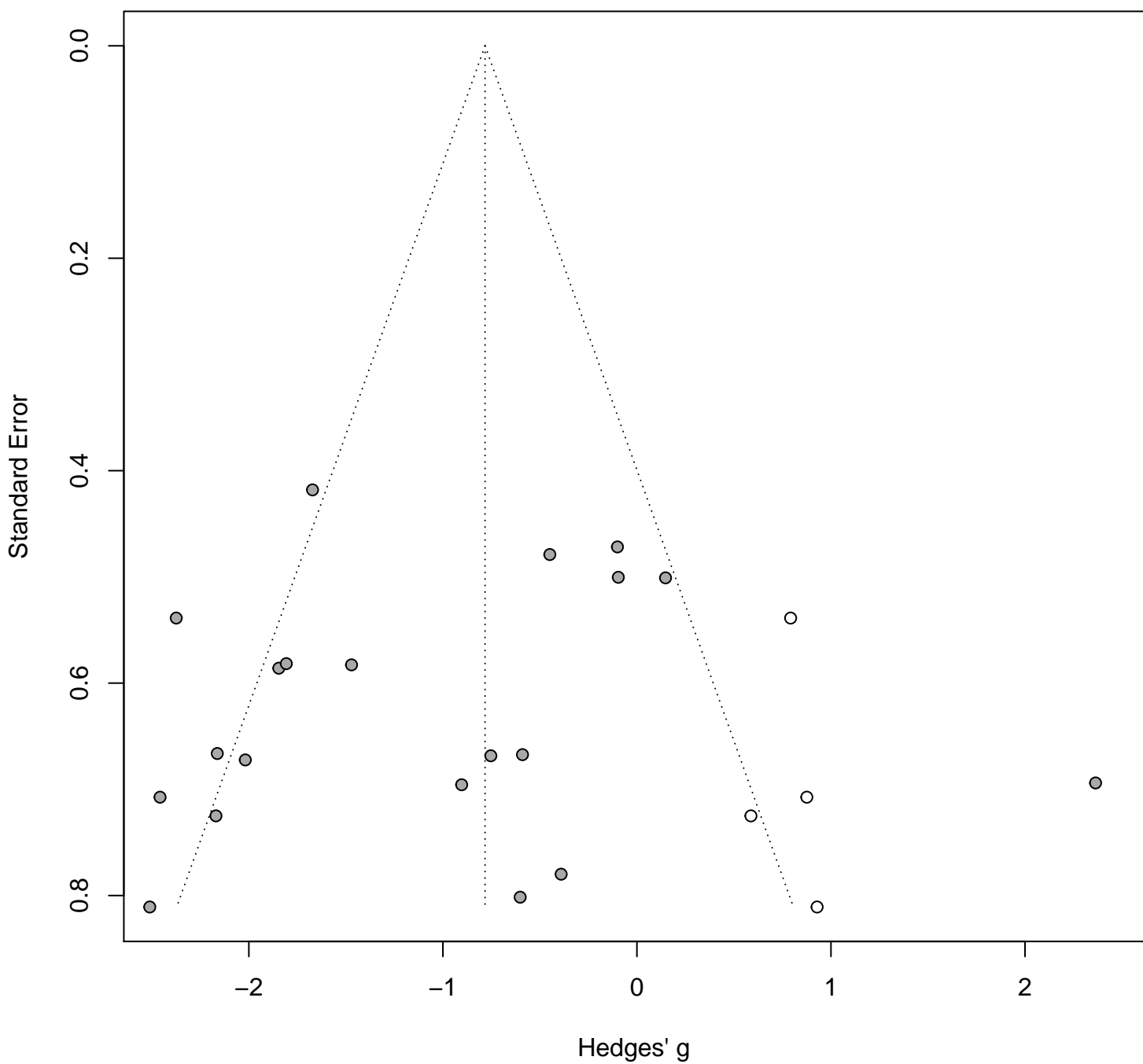

Supplement: Supplementary file 14 — Additional file 14: Figure S14. Funnel plot assessing for publication bias of CdM on right ventricular hypertrophy. [file 13287_2020_1900_MOESM14_ESM.pdf]

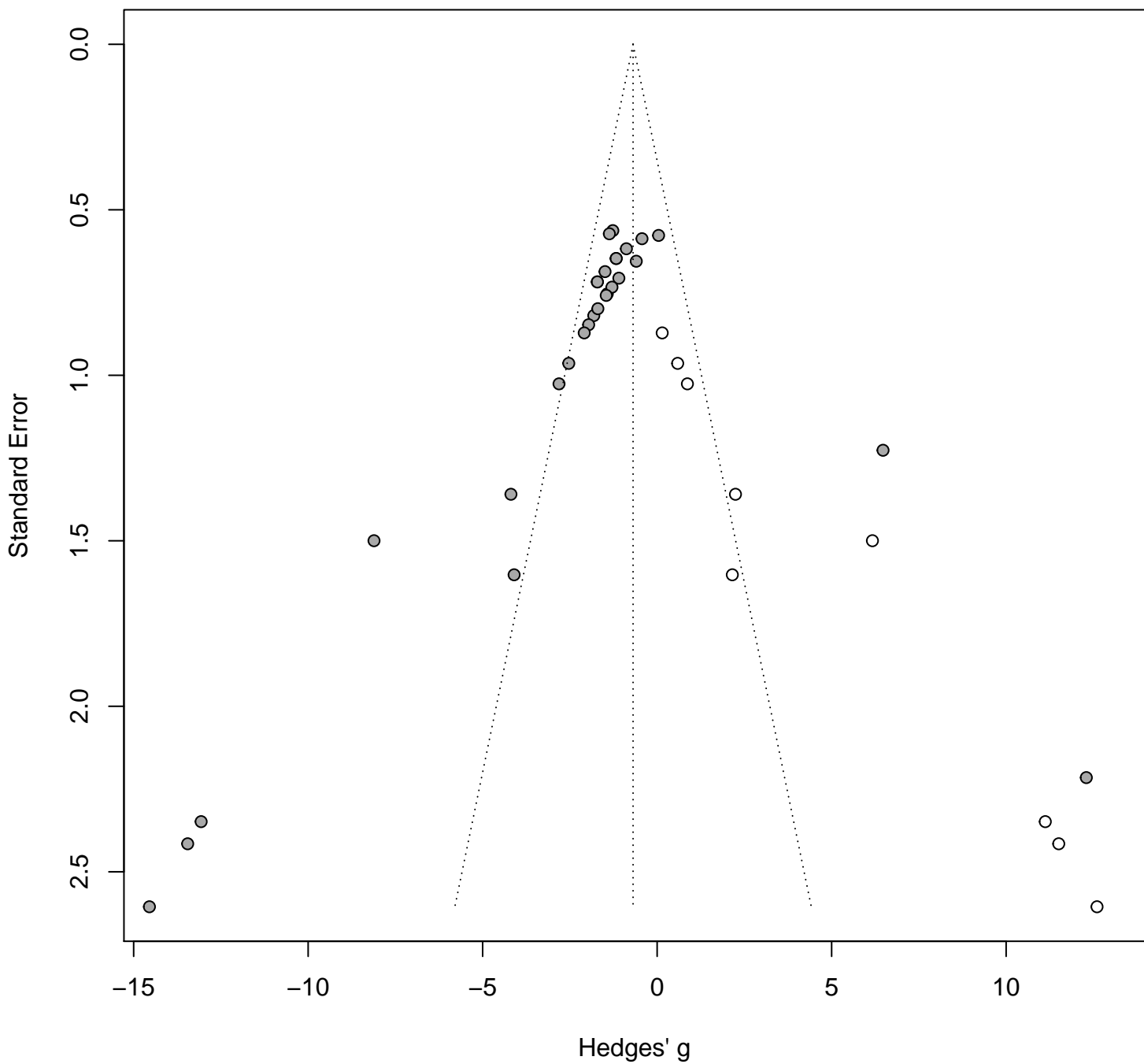

Supplement: Supplementary file 15 — Additional file 15: Figure S15. Funnel plot assessing for publication bias of CdM on lung fibrosis. [file 13287_2020_1900_MOESM15_ESM.pdf]

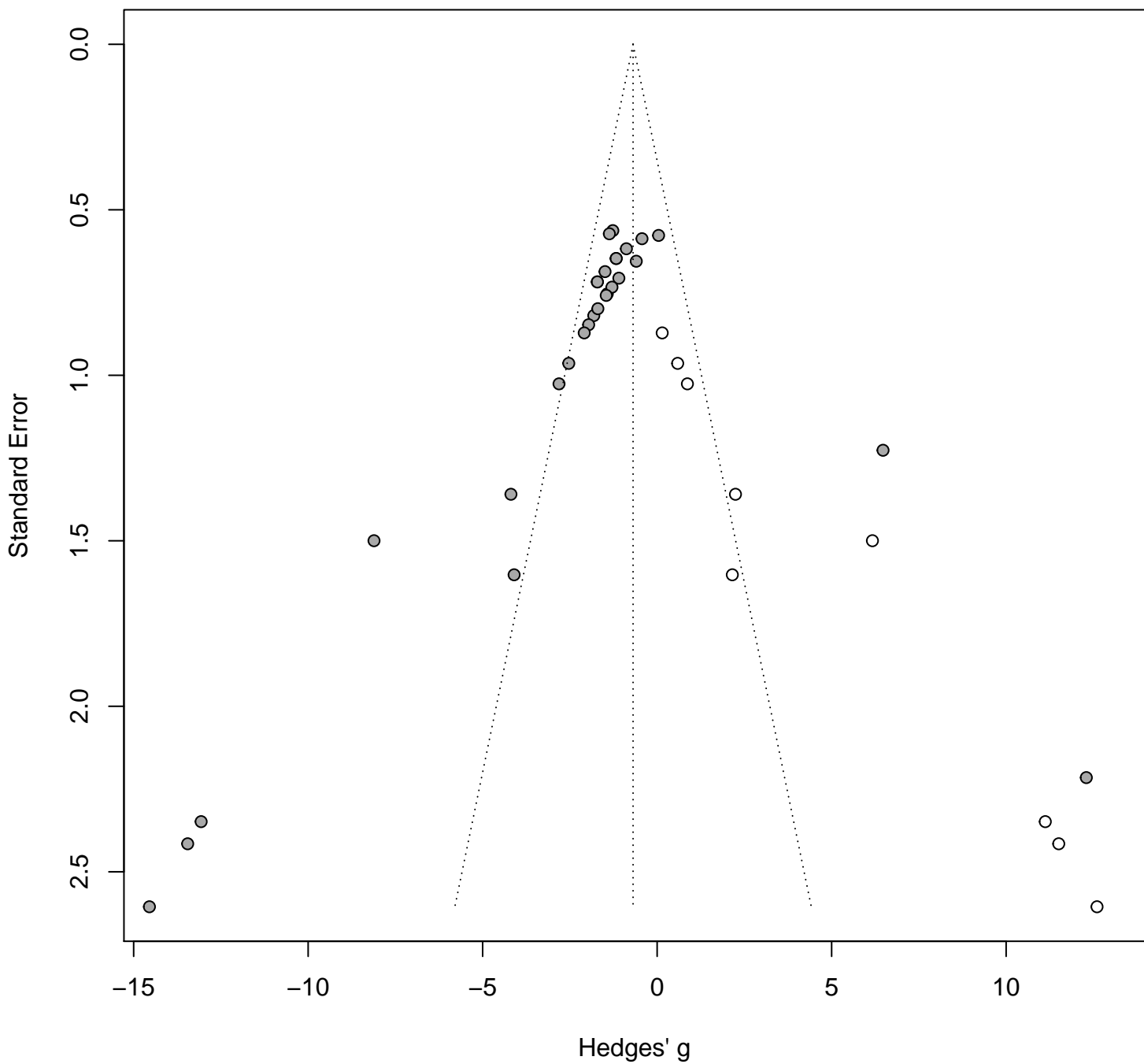

Supplement: Supplementary file 16 — Additional file 16: Figure S16. Funnel plot assessing for publication bias of CdM on pulmonary vasculogenesis. [file 13287_2020_1900_MOESM16_ESM.pdf]

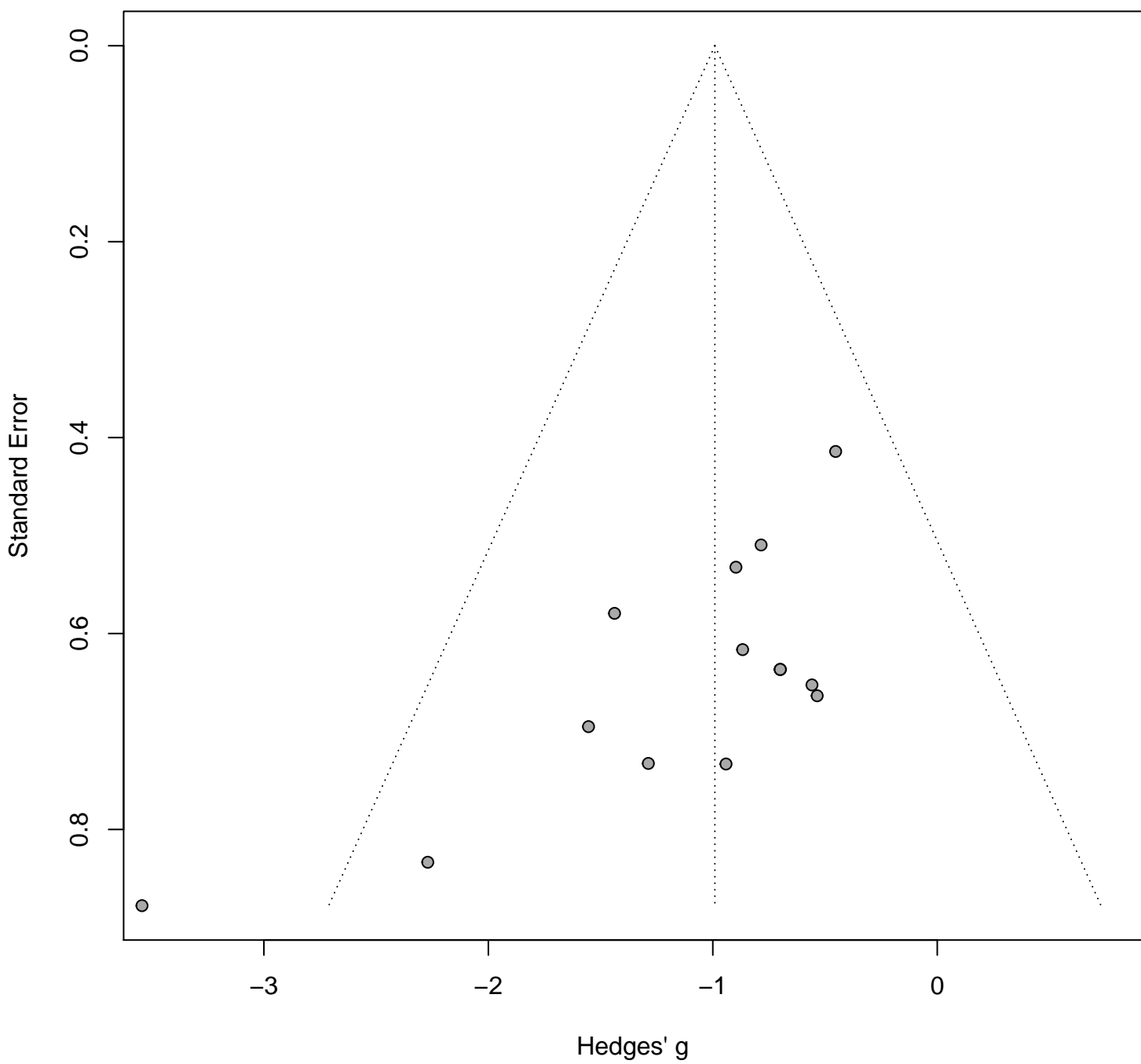

Supplement: Supplementary file 17 — Additional file 17: Figure S17. Funnel plot assessing for publication bias of CdM on lung permeability. [file 13287_2020_1900_MOESM17_ESM.pdf]

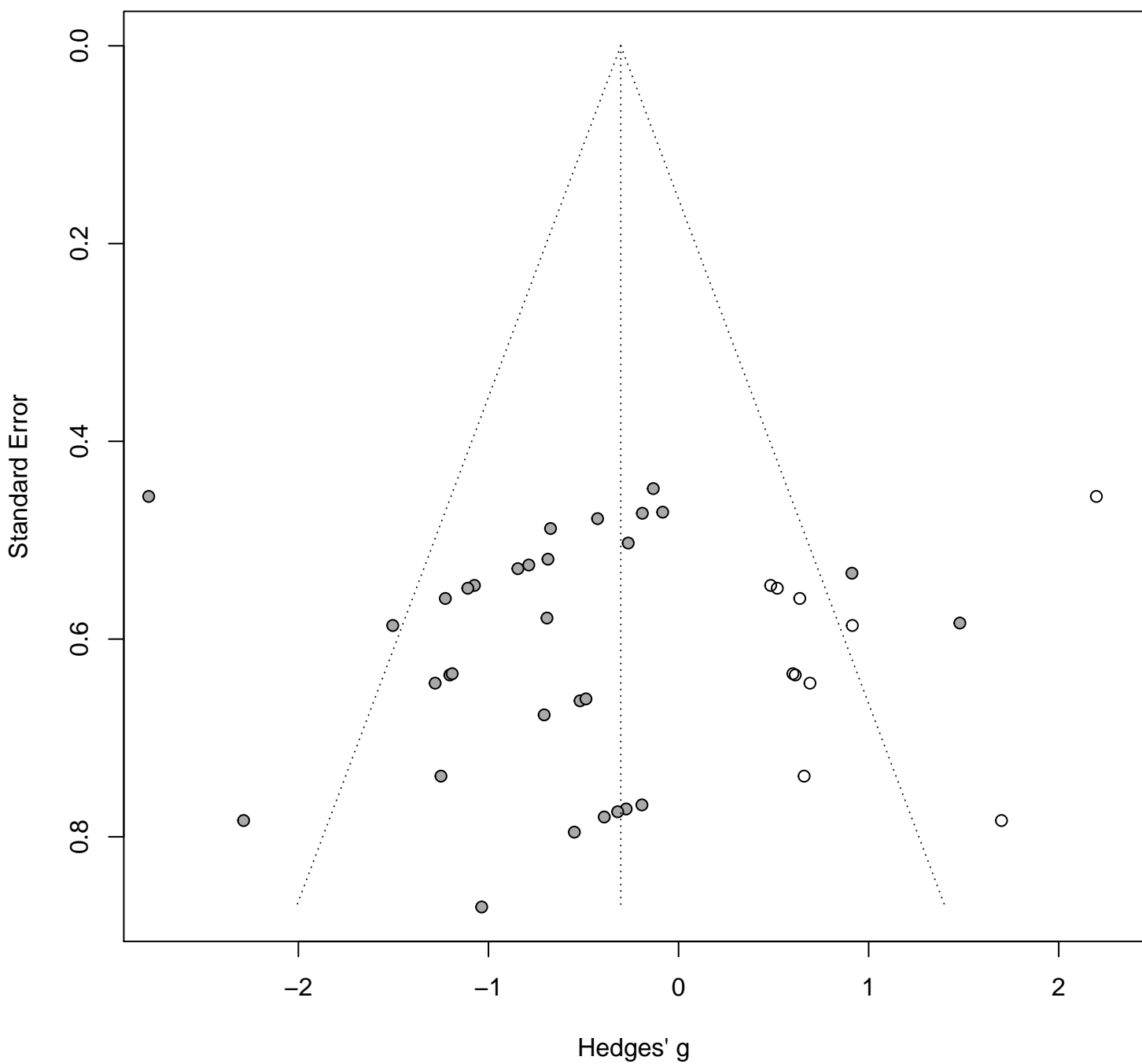

Supplement: Supplementary file 18 — Additional file 18: Figure S18. Funnel plot assessing for publication bias of CdM on pulmonary pressures. [file 13287_2020_1900_MOESM18_ESM.pdf]

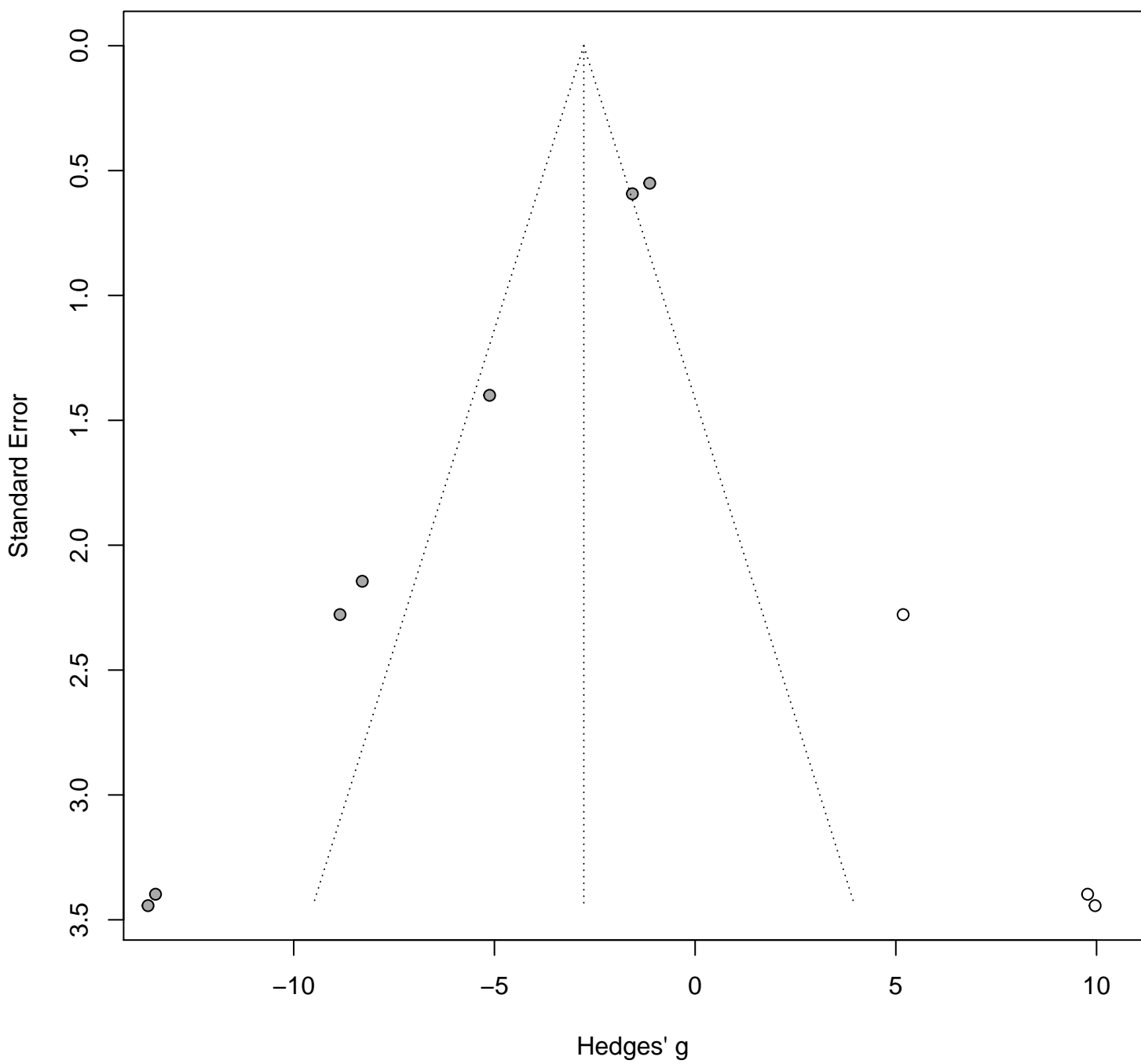

Supplement: Supplementary file 19 — Additional file 19: Figure S19. Funnel plot assessing for publication bias of CdM on histologic lung injury. [file 13287_2020_1900_MOESM19_ESM.pdf]

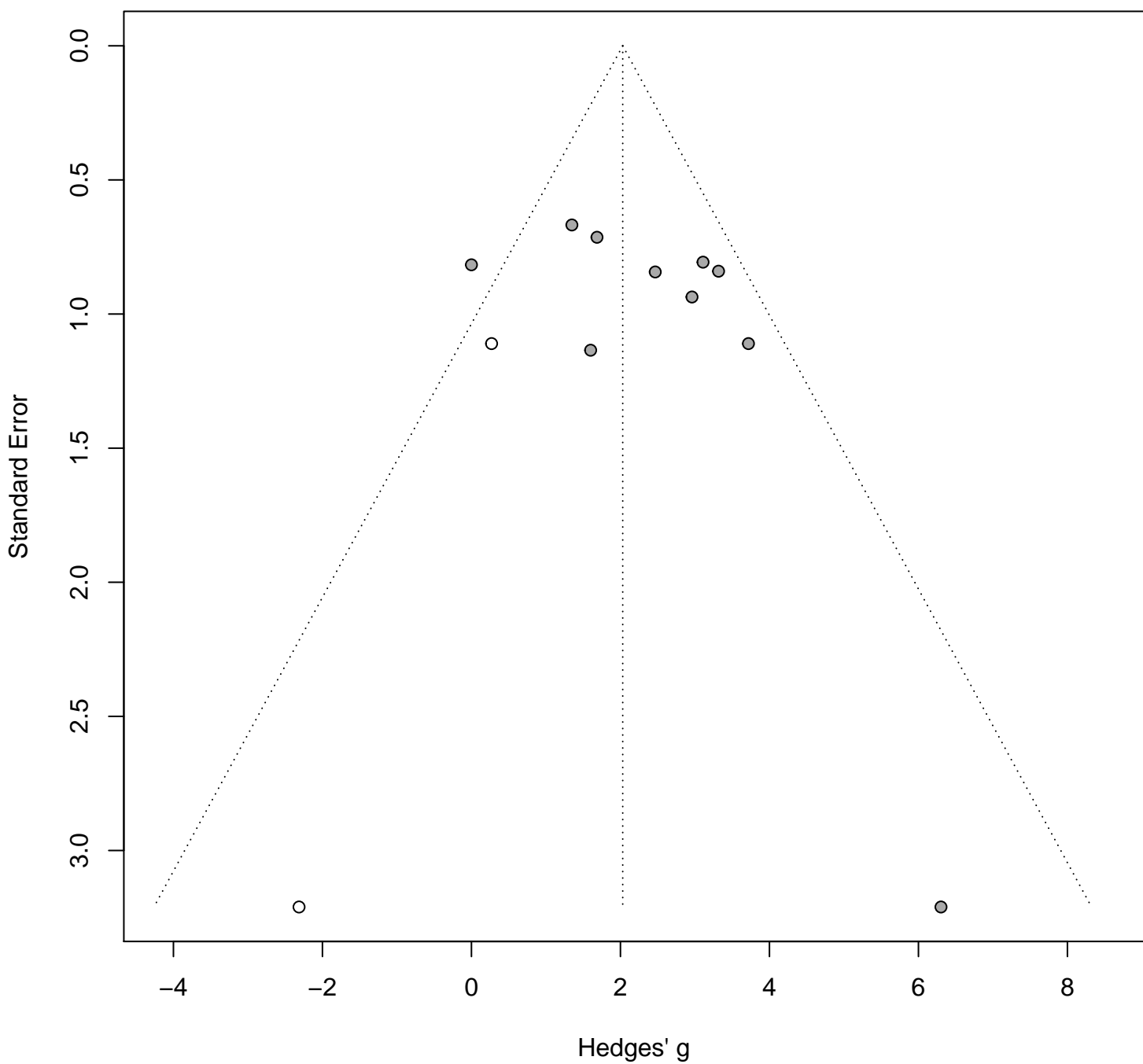

Supplement: Supplementary file 20 — Additional file 20: Figure S20. Funnel plot assessing for publication bias of CdM on lung compliance. [file 13287_2020_1900_MOESM20_ESM.pdf]
