# Supplementary material for: Effects of mesenchymal stromal cell-conditioned media on measures of lung structure and function: a systematic review and meta-analysis of preclinical studies
Source: Stem Cell Res Ther. 2020 Sep 15;11:399. doi: 10.1186/s13287-020-01900-7 (PMC7493362; doi:10.1186/s13287-020-01900-7)
Supplement: Supplementary file 21 — Additional file 21: File S1. List of articles included in this review. [file 13287_2020_1900_MOESM21_ESM.docx]

1. Ahmadi M, Rahbarghazi R, Soltani S, Aslani MR, Keyhanmanesh R. Contributory Anti-Inflammatory Effects of Mesenchymal Stem Cells, Not Conditioned Media, On Ovalbumin-Induced Asthmatic Changes in Male Rats. Inflammation. Inflammation; 2016;39:1960–71.

2. Ahmadi M, Rahbarghazi R, Aslani MR, Shahbazfar AA, Kazemi M, Keyhanmanesh R. Bone marrow mesenchymal stem cells and their conditioned media could potentially ameliorate ovalbumin-induced asthmatic changes. Biomed Pharmacother [Internet]. Elsevier Masson SAS; 2017;85:28–40. Available from: http://dx.doi.org/10.1016/j.biopha.2016.11.127

3. Aslam M, Baveja R, Liang OD, Fernandez-Gonzalez A, Lee C, Mitsialis SA, et al. Bone marrow stromal cells attenuate lung injury in a murine model of neonatal chronic lung disease. Am J Respir Crit Care Med. 2009;180:1122–30.

4. Chailakhyan RK, Aver’yanov A V., Zabozlaev FG, Sobolev PA, Sorokina A V., Akul’shin DA, et al. Comparison of the efficiency of transplantation of bone marrow multipotent mesenchymal stromal cells cultured under normoxic and hypoxic conditions and their conditioned media on the model of acute lung injury. Bull Exp Biol Med. 2014;157:138–42.

5. Chaubey S, Thueson S, Ponnalagu D, Alam MA, Gheorghe CP, Aghai Z, et al. Early gestational mesenchymal stem cell secretome attenuates experimental bronchopulmonary dysplasia in part via exosome-associated factor TSG-6. Stem Cell Res Ther. Stem Cell Research & Therapy; 2018;9:1–26.

6. Cruz F, Borg Z, Goodwin M, Sockocevic D, Wagner D, Coffey A, et al. Systemic administration of human bone marrow-derived mesenchymal stromal cell extracellular vesicles ameliorates Aspergillus hyphal extract-induced allergic airway inflammation in immunocompetent mice. Stem Cells Transl Med. 2015; 11:1302-16.

7. Curley GF, Ansari B, Hayes M, Devaney J, Masterson C, Ryan A, et al. Effects of intratracheal mesenchymal stromal cell therapy during recovery and resolution after ventilator-induced lung injury. Anesthesiology. 2013;118:924–33.

8. Felix RG, Bovolato ALC, Cotrim OS, Leão PDS, Batah SS, Golim M de A, et al. Adipose-derived stem cells and adipose-derived stem cell-conditioned medium modulate in situ imbalance between collagen I-and collagen V-mediated IL-17 immune response recovering bleomycin pulmonary fibrosis. Histol Histopathol. 2020;35:289–301.

9. Gülaşi S, Atici A, Yilmaz ŞN, Polat A, Yilmaz M, Laçin MT, et al. Mesenchymal stem cell treatment in hyperoxia-induced lung injury in newborn rats. Pediatr Int. 2016;58:206–13.

10. Hansmann G, Fernandez-Gonzalez A, Aslam M, Vitali SH, Martin T, Alex Mitsialis S, et al. Mesenchymal stem cell-mediated reversal of bronchopulmonary dysplasia and associated pulmonary hypertension. Pulm Circ. 2012;2:170–81.

11. Hayes M, Curley G, Masterson C, Devaney J, O’Toole D, Laffey J. Mesenchymal stromal cells are more effective than the MSC secretome in diminisheing injury and enhancing recovery following ventilator-induced lung injury. Intensive Care Medicine Experimental 2015;3:29.

12. Huh JW, Kim SY, Lee JH, Lee JS, van Ta Q, Kim M, et al. Bone marrow cells repair cigarette smoke-induced emphysema in rats. Am J Physiol - Lung Cell Mol Physiol. 2011;301:255–66.

13. Hwang B, Liles C, Waworuntu R, Mulligan M. Pretreatment with bone marrow derived mesenchymal stromal cell conditioned media confers pulmonary ischemic tolerance. J Thorac Cardiovasc Surg. 2016; 151:841-849.

14. Ionescu L, Byrne RN, van Haaften T, Vadivel A, Alphonse RS, Rey-Parra GJ, et al. Stem cell conditioned medium improves acute lung injury in mice: In vivo evidence for stem cell paracrine action. Am J Physiol - Lung Cell Mol Physiol. 2012;303:967–77.

15. Kennelly H, Mahon BP, English K. Human mesenchymal stromal cells exert HGF dependent cytoprotective effects in a human relevant pre-clinical model of COPD. Sci Rep [Internet]. Nature Publishing Group; 2016;6:1–11. Available from: http://dx.doi.org/10.1038/srep38207

16. Keyhanmanesh R, Rahbarghazi R, Aslani MR, Hassanpour M, Ahmadi M. Systemic delivery of mesenchymal stem cells condition media in repeated doses acts as magic bullets in restoring IFN-γ/IL-4 balance in asthmatic rats. Life Sci [Internet]. Elsevier; 2018;212:30–6. Available from: https://doi.org/10.1016/j.lfs.2018.09.049

17. Li X, An G, Wang Y, Liang D, Zhu Z, Tian L. Targeted migration of bone marrow mesenchymal stem cells inhibits silica-induced pulmonary fibrosis in rats. Stem Cell Res Ther. Stem Cell Research & Therapy; 2018;9:1–13.

18. Lu H, Poirier C, Cook T, Traktuev DO, Merfeld-Clauss S, Lease B, et al. Conditioned media from adipose stromal cells limit lipopolysaccharide-induced lung injury, endothelial hyperpermeability and apoptosis. J Transl Med. 2015;13:1–15.

19. Pierro M, Ionescu L, Montemurro T, Vadivel A, Weissmann G, Oudit G, et al. Short-term, long-term and paracrine effect of human umbilical cord-derived stem cells in lung injury prevention and repair in experimental bronchopulmonary dysplasia. Thorax. 2013;68:475–84.

20. Rahbarghazi R, Keyhanmanesh R, Aslani MR, Hassanpour M, Ahmadi M. Bone marrow mesenchymal stem cells and condition media diminish inflammatory adhesion molecules of pulmonary endothelial cells in an ovalbumin-induced asthmatic rat model. Microvasc Res [Internet]. Elsevier; 2019;121:63–70. Available from: https://doi.org/10.1016/j.mvr.2018.10.005

21. Rathinasabapathy A, Bruce E, Espejo A, Horowitz A, Sudhan DR, Nair A, et al. Therapeutic potential of adipose stem cell-derived conditioned medium against pulmonary hypertension and lung fibrosis. Br J Pharmacol. 2016;2859–79.

22. Sadeghi S, Mosaffa N, Hashemi SM, Mehdi Naghizadeh M, Ghazanfari T. The immunomodulatory effects of mesenchymal stem cells on long term pulmonary complications in an animal model exposed to a sulfur mustard analog. Int Immunopharmacol. 2020;80.

23. Shen Q, Chen B, Xiao Z, Zhao L, Xu X, Wan X, et al. Paracrine factors from mesenchymal stem cells attenuate epithelial injury and lung fibrosis. Mol Med Rep. 2015;11:2831–7.

24. Su V, Lin CS, Hung SH, Yang KY. Mesenchymal stem cell-conditioned medium induces neutrophil apoptosis associated with inhibition of the NF-κβ pathway in endotoxin-induced acute lung injury. Int J Mol Sci. 2019;20:2208.

25. Sutsko RP, Young KC, Ribeiro A, Torres E, Rodriguez M, Hehre D, et al. Long-term reparative effects of mesenchymal stem cell therapy following neonatal hyperoxia-induced lung injury. Pediatr Res. 2013;73:46–53.

26. Tropea KA, Leder E, Aslam M, Lau AN, Raiser DM, Lee JH, et al. Bronchioalveolar stem cells increase after mesenchymal stromal cell treatment in a mouse model of bronchopulmonary dysplasia. Am J Physiol - Lung Cell Mol Physiol. 2012;302:829–37.

27. Wakayama H, Hashimoto N, Matsushita Y, Matsubara K, Yamamoto N, Hasegawa Y, et al. Factors secreted from dental pulp stem cells show multifaceted benefits for treating acute lung injury in mice. Cytotherapy [Internet]. Elsevier Inc; 2015;17:1119–29. Available from: http://dx.doi.org/10.1016/j.jcyt.2015.04.009

28. Waszak P, Alphonse R, Vadivel A, Ionescu L, Eaton F, Thébaud B. Preconditioning enhances the paracrine effect of mesenchymal stem cells in preventing oxygen-induced neonatal lung injury in rats. Stem Cells Dev. 2012;21:2789–97.

29. Zhao, Y, Gillen J, Harris D, Kron I, Murphy M, Lau C. Treatment with placenta-derived mesenchymal cells mitigates development of bronchiolitis obliterans in a murine model. J Thorac Cardiovasc Surg. 2014;147:1668-1677.
