# Supplementary material for: Effects of mesenchymal stromal cell-conditioned media on measures of lung structure and function: a systematic review and meta-analysis of preclinical studies
Source: Stem Cell Res Ther. 2020 Sep 15;11:399. doi: 10.1186/s13287-020-01900-7 (PMC7493362; doi:10.1186/s13287-020-01900-7)
Supplement: Supplementary file 22 — Additional file 22: File S2. SYRCLE risk of bias. [file 13287_2020_1900_MOESM22_ESM.docx]

**Table 2.** SYRCLE Risk of Bias Assessment for included studies

| **No.** | **Author (Year)** | **Random sequence generation?** | **Groups similar at baseline?** | **Allocation concealed?** | **Animals randomly housed?** | **Blinding of caregivers and/or examiners?** | **Random selection for outcome assessment?** | **Blinding of outcome assessor?** | **Incomplete outcome data addressed?** | **Free from selective outcome reporting?** | **Free from other bias?** |
| --- | --- | --- | --- | --- | --- | --- | --- | --- | --- | --- | --- |
| 1 | Ahmadi (2017) | Yes | Yes | Unclear | Unclear | Unclear | Yes | Unclear | Yes | Yes | Yes |
| 2 | Ahmadi (2016) | Yes | Yes | Unclear | Unclear | Unclear | Unclear | Unclear | Yes | Yes | Yes |
| 3 | Aslam (2009) | Unclear | No | Unclear | Unclear | Unclear | Unclear | Unclear | Yes | Yes | Yes |
| 4 | Chailakhyan (2014) | Unclear | Yes | Unclear | Unclear | Unclear | Unclear | Unclear | Yes | Yes | Yes |
| 5 | Chaubey (2018) | Unclear | Yes | Unclear | Unclear | Unclear | Unclear | Yes | Yes | Yes | Yes |
| 6 | Cruz (2015) | Unclear | Yes | Unclear | Unclear | Unclear | Unclear | Unclear | Yes | No | Yes |
| 7 | Curley (2013) | Unclear | Yes | Unclear | Unclear | Unclear | Yes | Yes | Yes | Yes | Yes |
| 8 | Felix (2019) | Unclear | Yes | Unclear | Unclear | Unclear | Yes | Yes | Yes | Yes | Yes |
| 9 | Gulasi (2015) | Unclear | Yes | Unclear | Unclear | Unclear | Unclear | Yes | Yes | Yes | Yes |
| 10 | Hansmann (2012) | Unclear | Yes | Unclear | Unclear | Unclear | Unclear | Unclear | Yes | Yes | Yes |
| 11 | Hayes (2015) | Yes | Yes | Yes | Unclear | Unclear | Unclear | Unclear | Yes | Yes | Yes |
| 12 | Huh (2011) | Unclear | Yes | Unclear | Unclear | Unclear | Yes | Yes | Yes | Yes | Yes |
| 13 | Hwang (2016) | Unclear | No | Unclear | Unclear | Unclear | Unclear | Unclear | Yes | Yes | Yes |
| 14 | Ionescu (2012) | Unclear | Yes | Unclear | Unclear | Unclear | Unclear | Unclear | Yes | Yes | Yes |
| 15 | Kennelly (2016) | Unclear | Unclear | Unclear | Unclear | Unclear | Unclear | Unclear | Yes | Yes | Yes |
| 16 | Keyhanmanesh (2018) | Unclear | Yes | Unclear | Unclear | Unclear | Unclear | Unclear | Yes | Yes | Yes |
| 17 | Li (2018) | Yes | Yes | Unclear | Unclear | Unclear | Unclear | Unclear | Yes | Yes | Yes |
| 18 | Lu (2015) | Unclear | Yes | Unclear | Unclear | Unclear | Unclear | Unclear | Yes | Yes | Yes |
| 19 | Pierro (2012) | Yes | Unclear | Unclear | Unclear | Unclear | Unclear | Yes | Yes | Yes | Yes |
| 20 | Rahbarghazi (2019) | Yes | Yes | Unclear | Unclear | Unclear | Unclear | Unclear | Yes | Yes | Yes |
| 21 | Rathinasabapathy (2016) | Unclear | Yes | Yes | Unclear | Yes | Yes | Yes | Yes | Yes | Yes |
| 22 | Sadeghi (2019) | Yes | Yes | Unclear | Unclear | Unclear | Unclear | Yes | Yes | Yes | Yes |
| 23 | Shen (2014) | Yes | Yes | Unclear | Unclear | Unclear | Unclear | Unclear | Yes | Yes | Yes |
| 24 | Su (2019) | Unclear | Yes | Unclear | Unclear | Unclear | Unclear | Unclear | Yes | Yes | Yes |
| 25 | Sutsko (2012) | Unclear | No | Unclear | Unclear | Unclear | Yes | Yes | Yes | Yes | Yes |
| 26 | Tropea (2012) | Unclear | Yes | Unclear | Unclear | Yes | Yes | Yes | Yes | Yes | Yes |
| 27 | Wakayama (2015) | Unclear | Yes | Unclear | Unclear | Unclear | Yes | Unclear | Yes | Yes | Yes |
| 28 | Waszak (2012) | Unclear | Yes | Unclear | Unclear | Unclear | Unclear | Yes | Yes | Yes | Yes |
| 29 | Zhao (2014) | Yes | Yes | Unclear | Unclear | Unclear | Unclear | Yes | Yes | Yes | Yes |
