# Supplementary material for: Effects of mesenchymal stromal cell-conditioned media on measures of lung structure and function: a systematic review and meta-analysis of preclinical studies
Source: Stem Cell Res Ther. 2020 Sep 15;11:399. doi: 10.1186/s13287-020-01900-7 (PMC7493362; doi:10.1186/s13287-020-01900-7)
Supplement: Supplementary file 23 — Additional file 23: File S3. CdM characteristics. [file 13287_2020_1900_MOESM23_ESM.docx]

|  | **Supplementary File 2. CdM characteristics** | | | | | | | |
| --- | --- | --- | --- | --- | --- | --- | --- | --- |
| **No.** | **Author (Year)** | **Source** | **Were MSCs purchased or self-isolated?** | **Cell expansion media** | **Incubation time** | **Passage number** | **Dose** | **Notes** |
| 1 | Ahmadi (2016) | Rat bone marrow MSCs | Self-isolated | DMEM | 72 hours | 3 | 50 µl | Concentrated 50x; MW cutoff 4 kDa |
| 2 | Ahmadi (2017) | Rat bone marrow MSCs | Self-isolated | αMEM | 72 hours | 3 | 50 µl | Concentrated 50x; MW cutoff 4 kDa; concentration 500µg/mL |
| 3 | Aslam (2009) | Mouse bone marrow MSCs | Self-isolated | DMEM | 24 hours | 7-10 | 50 µl | Concentrated 10x; MW cutoff 10 kDa |
| 4 | Chailakhyan (2014) | Rat bone marrow MSCs | Self-isolated | NR | NR | 1-3 | 1000 µl | NR |
| 5 | Chaubey (2018) | Early gestational age human umbilical cord derived MSC | Self-isolated | DMEM-F12 | 24 hours | 3 | 100 μl | Concentrated 10x; MW cutoff 10 kDa |
| 6 | Cruz (2015) | Mouse bone marrow MSCs | Texas A&M stem cell core | IMDM | 24 hours | <7 | 200 µl | Concentrated 25x; MW cutoff 3 kDa |
| 7 | Curley (2013) | Rat bone marrow MSCs | Self-isolated | αMEM | 24 hours | 4 | 300 μl | 15 mls of medium was concentrated using a 3000 kDa centrifugal concentrating filter to give 300 μL |
| 8 | Felix (2020) | Adipose tissue MSCs | Self-isolated | DMEM | 24 hours | ≥3 | 200 µl | Concentrated 25x; cutoff 3 kDa |
| 9 | Gülaşı (2015) | Rat bone marrow MSCs | Self-isolated | NR | NR | NR | 25 μl | NR |
|  |  |  |  |  |  |  |  |  |
| 10 | Hansmann (2012) | Mouse bone marrow MSCs | Self-isolated | αMEM | 24 hours | 8-10 | 50 μl | Concentrated 10x; MW cutoff 10 kDa |
| 11 | Hayes (2015) | Rat femur and tibia MSCs | Self-isolated | αMEM | 24 hours | 4 | 500 µl | Concentrated 30x; MW cutoff 3 kDa |
| 12 | Huh (2011) | Rat bone marrow MSCs | Self-isolated | DMEM | 24 hours | 3-5 | 300 µl | Concentrated 20x; cutoff 3 kDa |
| 13 | Hwang (2016) | Rat bone marrow MSCs | Fred Hutchinson  Cancer Research Center | RPMI | NR | 6-12 | 200 µl | Centrifuged, 1200rpm, 10min |
| 14 | Ionescu (2012) | Mouse bone marrow MSCs | Self-isolated | DMEM | 24 hours | 2-8 | 30 µl | Concentrated 25x; MW cutoff 3 kDa |
| 15 | Kennelly (2016) | Human bone marrow | Self-isolated | DMEM | 24 hours | NR | NR | NR |
| 16 | Keyhanmanesh (2018) | Rat bone marrow MSCs | Self-isolated | DMEM/LG | 72 hours | 3-6 | 50 µl | Concentrated 50x; MW cutoff 4 kDa  500 μg/ml protein content |
| 17 | Li (2018) | Rat bone marrow MSCs | Self-isolated | αMEM | 24 hours | 3 | 1 mL | Cutoff 3 kDa |
| 18 | Lu (2012) | Human adipose MSCs | Self-isolated | EBM2 | 24 hours | 3 | 200 µl | Concentrated 10x; MW cutoff 3 kDa |
| 19 | Pierro (2012) | Human umbilical cord blood MSCs | Self-isolated | DMEM | 24 hours | NR | 7 μl/g | Concentrated 25x |
| 20 | Rahbarghazi (2019) | Rat bone marrow MSCs | Self-isolated | DMEM/LG | 72 hours | 3-6 | 50 µl | Concentrated 50x; MW cutoff 4 kDa  500 μg/ml protein content |
| 21 | Rathinasabapathy (2012) | Rat adipose MSCs | Self-isolated | DMEM | 24 hours | 2-5 | 100 µl | Concentrated to dose; MW cutoff 3 kDa |
| 22 | Sadeghi (2019) | Mouse adipose MSCs | Self-isolated | DMEM-F12 medium | 24 hours | 2 | 500 μl | NR |
| 23 | Shen (2014) | Rat bone marrow MSCs | Self-isolated | DMEM | 24 hours | 3 and 4 | 200 µl | Concentrated 25x; MW cutoff 3-kDa |
| 24 | Su (2019) | Mouse MSC | Self-isolated | NR | 48 hours | NR | 200 µl | Concentrated 50x; MW cutoff 5-kDa |
| 25 | Sutsko (2012) | Rat bone marrow MSCs | Self-isolated | αMEM | 24 hours | NR | 50 µl | NR |
| 26 | Tropea (2012) | Mouse bone marrow MSCs | Self-isolated | αMEM | 24 hours | NR | 50 μl | Concentrated 10x; MW cutoff 10 kDa |
| 27 | Wakayama (2015) | Human teeth MSCs | Self-isolated | DMEM | 48 hours | 3-9 | 500 µl | Average protein [3 µg/mL] |
| 28 | Waszak (2012) | Rat bone marrow MSCs | Self-isolated | DMEM | 24 hours | 2 | 1 µl/g | Concentrated 25x; MW cutoff 3 kDa |
| 29 | Zhao (2014) | Mouse Placenta-derived MSCs | Self-isolated from collaborator | DMEM/F12K (1:1) | NR | NR | NR | NR |

DMEM-Dulbecco’s Modified Eagle Medium; EBM-endothelial basal medium; LG-low glucose; MSC-mesenchymal stromal cell; MW-molecular weight; NR-not reported; rpm-revolutions per minute; RPMI- Roswell Park Memorial Institute
